# Structure–Function Analysis of the Essential *Mycobacterium tuberculosis* P450 Drug Target, CYP121A1

**DOI:** 10.3390/ijms25094886

**Published:** 2024-04-30

**Authors:** Tiara Padayachee, David C. Lamb, David R. Nelson, Khajamohiddin Syed

**Affiliations:** 1 Department of Biochemistry and Microbiology, Faculty of Science, Agriculture and Engineering, University of Zululand, Empangeni 3886, South Africa; teez07padayachee@gmail.com; 2 Faculty of Medicine, Health and Life Sciences, Swansea University, Swansea SA2 8PP, UK; d.c.lamb@swansea.ac.uk; 3 Department of Microbiology, Immunology and Biochemistry, University of Tennessee Health Science Center, Memphis, TN 38163, USA; drnelson1@gmail.com

**Keywords:** cytochrome P450, *Mycobacterium tuberculosis*, CYP121A1, cYY, active site, crystal structure, amino acid residues, heme

## Abstract

Cytochrome P450 CYP121A1 is a well-known drug target against *Mycobacterium tuberculosis*, the human pathogen that causes the deadly disease tuberculosis (TB). CYP121A1 is a unique P450 enzyme because it uses classical and non-classical P450 catalytic processes and has distinct structural features among P450s. However, a detailed investigation of CYP121A1 protein structures in terms of active site cavity dynamics and key amino acids interacting with bound ligands has yet to be undertaken. To address this research knowledge gap, 53 CYP121A1 crystal structures were investigated in this study. Critical amino acids required for CYP121A1’s overall activity were identified and highlighted this enzyme’s rigid architecture and substrate selectivity. The CYP121A1-fluconazole crystal structure revealed a novel azole drug–P450 binding mode in which azole heme coordination was facilitated by a water molecule. Fragment-based inhibitor approaches revealed that CYP121A1 can be inhibited by molecules that block the substrate channel or by directly interacting with the P450 heme. This study serves as a reference for the precise understanding of CYP121A1 interactions with different ligands and the structure–function analysis of P450 enzymes in general. Our findings provide critical information for the synthesis of more specific CYP121A1 inhibitors and their development as novel anti-TB drugs.

## 1. Introduction

Globally, tuberculosis (TB) caused an estimated 1.3 million deaths in 2022, including 167,000 people with Human Immunodeficiency Virus (HIV), thus making TB the second leading infectious disease after coronavirus disease (COVID-19) [1]. *Mycobacterium tuberculosis* is the pathogen responsible for this deadly lung disease in humans. Additionally, 7.5 million new tuberculosis cases were reported worldwide in 2022 [1]. This is the highest number of reported TB cases since the World Health Organization (WHO) started monitoring global TB infections in 1995, surpassing the pre-COVID peak of 7.1 million in 2019 and increasing from 5.8 million in 2020 to 6.4 million in 2021 [1]. Without treatment, the death rate from TB disease is high (about 50% mortality) [2]. Worldwide, an estimated 410,000 people developed rifampicin-resistant (rifampicin is the most powerful first-line anti-TB drug) (RR-TB) or multidrug-resistant (resistance to rifampicin and isoniazid) (MDR-TB) tuberculosis in 2022 [1], indicating the urgent need for developing new anti-TB drugs with a novel mode of action to combat this pathogen.

Analysis of the *M. tuberculosis* H37Rv genome sequence revealed the presence of 20 cytochrome P450 monooxygenases (CYPs/P450s) encoded in its genome [3]. P450s are a superfamily of heme-containing enzymes found in species across the domains of life [4]. Many P450s catalyze key enzymatic reactions in cells with strict stereo- and regio-specificity and, hence, are drug targets of interest when encoded in pathogens [5,6]. Studies revealed that three P450s, namely, CYP121, CYP125, and CYP142, are potential drug targets in *M. tuberculosis* [7]. Gene-knock-out studies indicated that CYP121 is essential for the viability of *M. tuberculosis* H37Rv [8].

**Figure 1:**
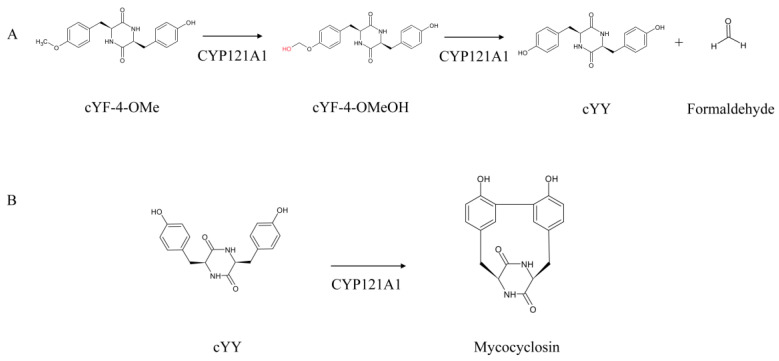
Functional analysis of CYP121A1 from *Mycobacterium tuberculosis* H37Rv. (**A**) CYP121 hydroxylation of cyclo(L-Tyr-L-Phe-4-OMe) (cYF-4-OMe) to form cyclo(L-Tyr-L-Phe-4-OMeOH) (cYF-4-OMeOH) followed by demethylation of cyclo(L-Tyr-L-Phe-4-OMeOH) to form cYY with the release of formaldehyde [9]. (**B**) CYP121 carbon-carbon coupling of cyclo-(L-Tyr-L-Tyr) (cYY) to produce mycocyclosin [10].

CYP121A1 hydroxylates cyclo(L-Tyr-L-Phe-4-OMe) to form cyclo(L-Tyr-L-Phe-4-OMeOH) followed by demethylation of cyclo(L-Tyr-L-Phe-4-OMeOH) to form cyclo-(L-Tyr-L-Tyr) with the elimination of formaldehyde (Figure 1A) [9]. Interestingly, the biological function of CYP121A1 was identified based on its downstream association with an adjacent gene, Rv2275, forming an operon structure [10]. The Rv2275 gene product is involved in the synthesis of the cyclodipeptide cyclo(L-Tyr-L-Tyr) (cYY) [11]. CYP121A1 was found to catalyze the oxidative coupling of two tyrosine residues in cYY, resulting in the formation of mycocyclosin (Figure 1B) [10]. Based on this functional analysis, it is clear that CYP121A1 is unique among P450s as it can catalyze the C-C coupling reaction, C-H bond activation, hydroxylation of a methyl group, and *O*-demethylation as a single enzyme. A comprehensive analysis of P450 sequences in 2666 *Mycobacterium* species revealed that CYP121, CYP124 and CYP128 are all part of the same biosynthetic gene cluster, indicating that these three P450s have a collective role in synthesizing complex natural metabolites that play an important role in the mycobacterial species [12,13].

Previous structural analysis of CYP121A1 revealed novel features among P450s. The overall structural architecture of CYP121A1 was found to be highly rigid, and negligible dynamic changes were observed upon the binding of ligands [8,14]. The CYP121 heme orientation was present in two distinct conformations related by 180° rotation around an axis of symmetry [14]. Catalytically, CYP121A1 was found not to follow the classical P450 catalytic cycle mechanism during cYY C-C coupling; instead, it follows a peroxidase-like mechanism involving the generation of substrate diradical with the sixth water ligand of heme iron never displaced [15,16]. Among all *M. tuberculosis* P450s characterized, CYP121A1 was found to bind to azole drugs, known P450 inhibitors, tightly [7,17]. CYP121A1 interactions with fluconazole revealed a novel azole–P450 binding mode [18]. Many studies have focused on identifying potential inhibitors against CYP121A1 as a route to develop novel anti-TB drugs.

Recently, our comprehensive analysis of 44 CYP107 crystal structures revealed critical new information about CYP107 active site amino acid residues and their dynamics and highlighted the multi-functionality of this P450 family [19]. Such work paves the way for the genetic engineering of CYP107s, which can help in the production of novel molecules of biotechnological interest. CYP121 is a novel P450 drug target in *M. tuberculosis*. CYP121 displays unique features amongst all P450s, and the availability of numerous CYP121 crystal structures with bound inhibitors at the Research Collaboratory for Structural Bioinformatics Protein Data Bank (RCSB PDB) [20] allows, for the first time, for a comprehensive analysis of CYP121A1 structure–activity relationships focusing on active site cavity dynamics and key amino acids playing a role in interacting with ligands (substrate or inhibitors). In this study, we address this research knowledge gap. We analyzed 53 CYP121 crystal structures and delineated the structure–function relationships of CYP121A1, including the nature of this P450, catalytic mechanisms, and carried out a comprehensive analysis of its interactions with ligands (substrate and inhibitors). This work contributes to the effort to develop specific CYP121 inhibitors to treat *M. tuberculosis* infections.

## 2. Results and Discussion

### 2.1. CYP121A1 Is a Highly Rigid Enzyme

The majority of CYP121A1 crystal structures characterized were found to be in a closed conformation (83%) (Table S1). This was expected as this P450, a drug target, is crystallized in a complex with numerous ligands. The area for CYP121 open conformations ranged between 1163 Å^2^ and 1215 Å^2^, and the volume ranged between 722 Å^3^ and 782 Å^3^. In contrast, the closed conformation area ranged between 993 Å^2^ and 1228 Å^2^, and the volume ranged between 690 Å^3^ and 838 Å^3^ (Table S1). Using the available crystal structures data, the average area and volume for CYP121A1 in an open conformation is 1174 Å^2^ and 751 Å^3^ compared to 1137 Å^2^ and 743 Å^3^ for a closed conformation. Therefore, the estimated change in area and volume from the open to closed conformation is 37 Å^2^ and 8 Å^3^, reflecting the decrease in the area and volume from open to closed conformation. However, the change from open to closed conformation is almost negligible when compared to the other P450s, such as the CYP107 family, where the change in area and volume from open to closed conformation is 276 Å^2^ and 494 Å^3^, respectively [19]; CYP3A4 has a change in volume from open to closed conformation is 1173 Å^3^ to 2017 Å^3^ when bound to ketoconazole, and 2682 Å^3^ when bound to erythromycin [21]; and CYP2A6 has a change in volume from open to closed conformation is 251 Å^3^ to 300 Å^3^ when bound to phenacetin) [22]. These findings strongly indicate that CYP121A1 is a rigid enzyme. Molecular dynamic analysis of amino acids for 53 CYP121A1 crystal structures revealed a lowest root-mean-square difference (RMSD) of 0.225 Å compared to CYP107FH5 (RMSD of 3.0 Å), further strongly supporting the idea of the rigid nature of CYP121A1.

Analysis of CYP121A1 active site cavity amino acids revealed that all the amino acids that form the open conformation (36 amino acids) are also present in the closed conformation, indicating their key role in maintaining the active site’s architecture (Table 1). In open conformation crystal structures, out of 36 amino acids, 33 amino acids were conserved. In closed conformation crystal structures, out of 47 amino acids, only 27 were conserved, indicating the dynamic nature of amino acids of CYP121A1 during interactions with different ligands. Eleven amino acids were unique to closed conformation, not conserved in CYP121As (Table 1).

### 2.2. Active Site Analysis Revealed Unusual Features and Key Amino Acids Governing the Structure and Function of CYP121A1

Analysis of the CYP121A1 open conformation structure revealed novel aspects of the P450 structure [14]. The heme was bound in two distinct conformations rotated 180° through an axis of symmetry. The macrocycle of the heme was distorted due to the proline side chains’ interactions with one of its pyrrole groups [14]. The active site was relatively rigid as the amino acid residues above the heme plane were involved in a hydrogen-bonding network that determined the active site morphology. Furthermore, these residues are involved in the proton relay system [8,14].

Active site analysis of CYP121A1 revealed the presence of critical amino acid residues involved in various processes, such as the maintenance of active site structure (Ala233, Ser237, and Arg386), hydrogen bonding and proton relay systems (Ser237, Arg386, and Ser279), heme orientation and water ligand retention (Pro346, Ser237, and Arg386), and thermodynamic regulation of heme iron (Phe338) [14]. These residues were mutated and analyzed to further understand these amino acids’ roles in CYP121 structure and azole binding.

**Figure 2:**
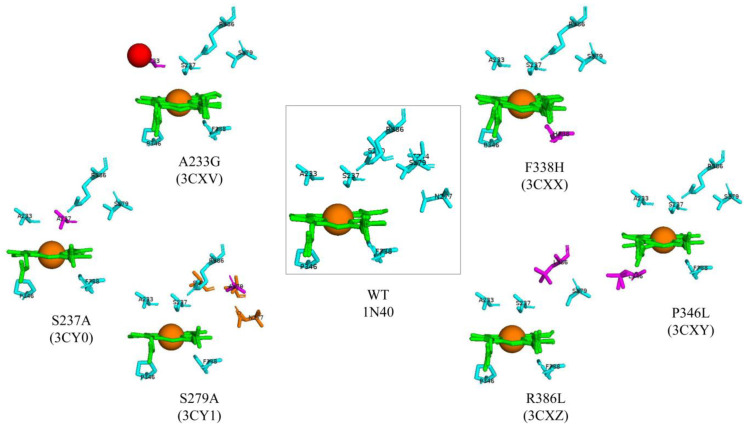
Active site analysis of CYP121A1 wild-type (WT) and six mutants. CYP121A1-WT is shown in the box. WT amino acids are colored in cyan. Heme is shown in green, the iron atom is shown as a brown sphere, a water molecule is shown as a red sphere, mutated amino acids are shown in magenta, and conformational changed amino acids are shown in orange. Amino acid residues are labeled according to their single-letter codes. The PDB code is shown in brackets underneath the respective mutant.

CYP121A1 mutants used for the in silico analysis were A233G, F338H, S237A, S279A, R386L, and P346L. All the mutants maintained the same P450 conformation as the wild-type (WT) protein [8] (Figure 2). However, the P346L mutation had significant effects on the conformation of the heme orientation. In the WT structure, the Pro346 side chain was in close contact with the heme and distorted the plane [14]. Removing this side chain in the P346L mutant led to a more planar heme conformation (Figure 2).

A233G and S237A mutants significantly altered the environment of the CYP121A1 heme sixth ligand. A water molecule in the A233G mutant structure occupied the same position as Ala233 in the WT [14] (Figure 2). UV–visible spectroscopic analysis of the CYP121A1 A233G mutant revealed that the additional water molecule in the vicinity of the heme iron was an alternative position for the sixth water ligand, and the A233G distal water was in an equilibrium between these positions. This resulted in a shift towards a high spin [8]. The heme sixth ligand was closer to the Ala233 carboxyl backbone in the S237A mutant (Figure 2). This led to a considerable increase in the distance between the heme iron and distal water.

Three CYP121A1 amino acid residues were postulated to be involved in a proton relay system: Asn277, Ser240, and Thr244 [14]. The conformations of these amino acid residues were altered in the S279A mutant (Figure 2). These changes altered the hydrogen bond network within the CYP121A1 active site [8], thus highlighting the importance of the Ser279 proton relay system. Experimental studies based on the wild type of CYP121A1 revealed a negative reduction potential for the heme iron [14]. Spectro-electrochemical titration studies revealed that the redox potentials of the majority of the mutants did not significantly change. However, a slight increase in heme iron reduction potential was observed for the A233G and S237A mutants [8]. Interestingly, despite the planarity of the heme in the P346L mutant, there was no significant change in the heme potential, thus indicating that the distortion does not substantially regulate CYP121A1 heme iron potential [8]. Heme potential was not perturbed in the F338H mutant, suggesting that the role of phenylalanine in heme thermodynamic regulation is unlikely. In contrast, a significant change in redox potential was observed for the R386L mutant [8]. This change resulted in an almost completely low-spin P450 form. This indicates the critical nature of Arg386 in CYP121A1 thermodynamic regulation. Replacement of arginine or removal of its charge has a significant impact on triggering CYP121A1 activity by thermodynamically favoring electron transport from redox partners [8].

### 2.3. The Binding of cYY Reveals Unique Structural Aspects of CYP121A1

**Figure 3:**
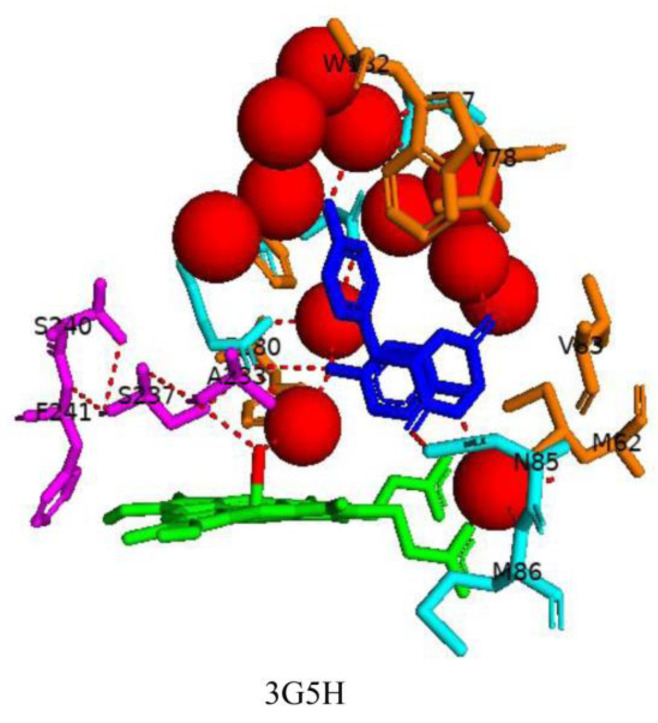
Analysis of CYP121A1 interactions with its substrate, cyclo(L-Tyr-L-Tyr) (cYY). Heme is shown in green; the sixth water ligand is shown as a red stick; the substrate is blue; and amino acids involved in a hydrogen-bonded network are shown in magenta. Amino acid residues sharing a direct and water-mediated polar interaction with the substrate are shown in cyan, and amino acid residues sharing van der Waals interactions with the substrate are shown in orange. Polar interactions are indicated as red dashed lines, water molecules are represented as red spheres, and amino acid residues are labeled according to their single-letter codes. PDB code is shown underneath the respective model. A list of all amino acid residues within 5 Å of the substrate is shown in Table 2.

cYY binds comfortably within the binding pocket of CYP121A1 (Figure 3). The diketopiperazine (DKP) ring adopts a flattened boat conformation, and one tyrosyl side chain faces the ring. CYP121A1 active site contains unique water pockets. cYY interacts with two defined water pockets within the active site (Figure 3). The binding of cYY in the CYP121A1 active site involves multiple van der Waals contacts with hydrophobic side chains of amino acid residues: Met-62, Val-78, Val-83, Phe-168, Trp-182, Ala-233, and Phe-280 [10]. One carbonyl of the DKP ring forms a direct hydrogen bond with a nitrogen atom of Asn85 (Figure 3). The substrate also forms direct hydrogen bonds with five water molecules, leading to water-mediated polar interactions with six additional water molecules (Figure 3). The hydroxyl group of one tyrosyl moiety forms a water-mediated bond with the oxygen atom of Thr77, and the nitrogen atom of the DKP ring forms a water-mediated bond with the nitrogen atom of Met86 and one propionate group of the heme [10]. The hydroxyl group closest to the heme interacts via a water molecule with the sixth water ligand and forms water-mediated bonds with the nitrogen of Gln385 and Arg386 (Figure 3).

Substrate binding in P450s usually results in the displacement of the sixth water ligand; after that, the iron becomes penta-coordinated and is in a high spin state [23]. Interestingly, the sixth water ligand was not displaced upon cYY binding as previously observed when fluconazole binds (see below) [18]. As mentioned in Section 2.2, an unusual hydrogen bonding network occurs above the heme in the CYP121A1 active site [14]. This network is also present upon cYY binding, and the sixth water ligand actively participates in this network (Figure 3). The hydroxyl group of Ser237 interacts with the sixth water ligand, Ala233, and the nitrogen atom of Arg386. The oxygen atom of Ser237 interacts with Ser240 and Phe241. Ser237 and Arg386 are postulated to be involved in the hydrogen network, and these results further support this theory. Interestingly, no significant structural changes were observed between the ligand-free and cYY-bound crystal structures of CYP121A1 [8,10]. This further supports the notion that CYP121A1 is a rigid enzyme, as discussed in Section 2.1.

### 2.4. CYP121A1 Performs Novel Catalytic Reactions

#### 2.4.1. C-C Intramolecular Coupling

**Figure 4:**
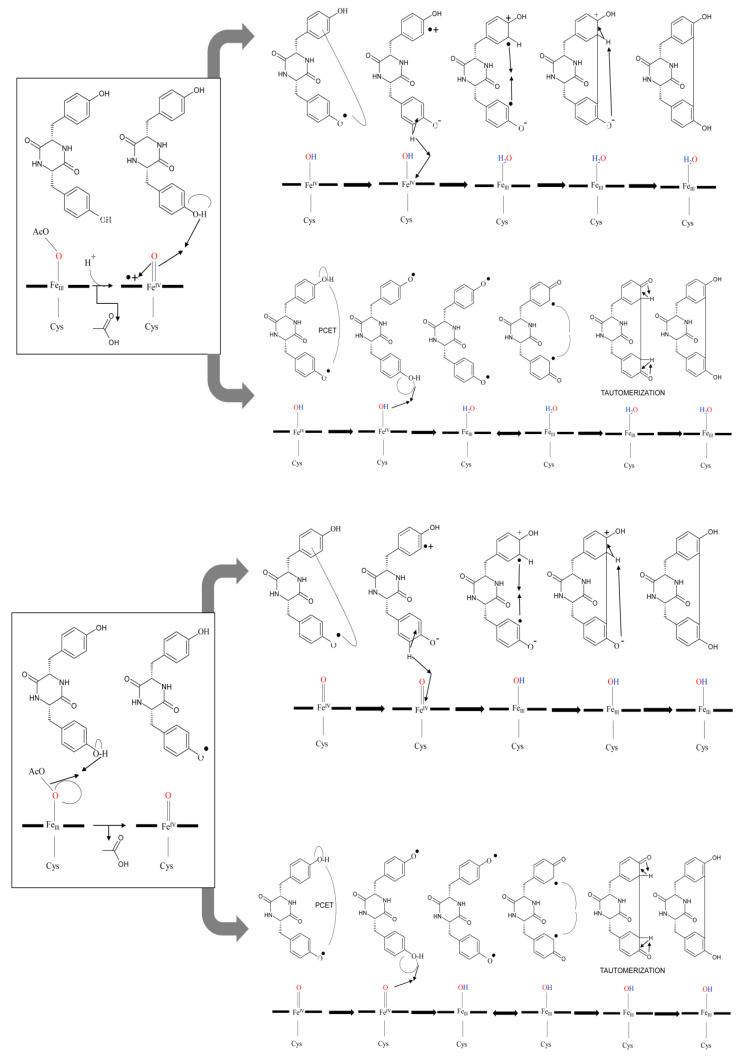
Schematic representation of the four suggested mechanistic pathways for the C-C coupling reaction of cYY performed by CYP121A1 using peracetic acid as an oxidant [15]. The figure is adapted from Ref. [15].

CYP121A1 does not follow the classical P450 enzymatic reaction mechanism as it does not generate the iron-bound oxidant; instead, it performs a peroxidase-type reaction, generating the substrate-based radicals and does not displace the sixth water ligand from heme iron during the catalytic reaction [15,16]. CYP121A1 performs an unusual C-C coupling reaction involving the oxidizing species, compound Ι (Fe(IV)+•=O), a ferric peroxo, or both [15]. Analysis of the catalytic mechanism of CYP121A1 with peracetic acid revealed four potential mechanistic pathways (Figure 4) [15]. Compound Ι performs a hydrogen abstraction of the hydroxyl group of the proximal tyrosine moiety of cYY, resulting in a protonated compound II (Fe(IV)-OH) and a substrate radical (Figure 4). Subsequently, this reaction can follow two paths; the reaction can proceed via an electron tunnelling step to produce a cation radical and phenolate chemical species followed by a second hydrogen atom abstraction at the distal position of the tyrosine moiety facilitated by compound II (Fe(IV)-OH) (Figure 4) [15]. This step generates the second radical on cYY, and the two opposite-charged radicals on cYY will rapidly combine to produce a new C–C bond (Figure 4) [15]. Deprotonation of the distal tyrosine moiety by the phenolate species will re-aromatize the product and form mycocyclosin (Figure 4). This reaction can also take another route by replacing the electron tunnelling step with a proton-coupled electron transfer step (PCET) (Figure 4) [15]. Here, the radical from the proximal tyrosine moiety will migrate to the distal tyrosine moiety (Figure 4). A second hydrogen atom abstraction will occur between Fe(IV)-OH and the hydroxyl moiety of the proximal tyrosine, forming the desired diradical substrate (Figure 4) [15]. The two radicals are delocalized on the aromatic ring, which allows the diradical to combine and form the C-C bond. Tautomerization of this compound will then produce mycocyclosin (Figure 4) [15]. Both pathways are proposed where compound I is the likely oxidizing species.

However, a ferric peroxo species (Ac-O-OFe(III)) can directly oxidize cYY, resulting in an oxoferryl species and a substrate radical (Figure 4) [15]. Consequently, two reaction paths can be taken to form the C-C bond and mycocyclosin. The first pathway involves a direct electron tunnelling system via a protein amino acid and the radical on the proximal tyrosine moiety (Figure 4) [15]. Oxidation of the proximal tyrosine amino acid generates the diradical substrate required for the radical–radical reaction, forming the C-C bond formation (Figure 4) [15]. The electron tunnelling system can be replaced with a proton-coupled electron transfer step where the high valent iron group can oxidize the hydroxyl group on the proximal tyrosine moiety, resulting in the diradical substrate followed by the rapid C-C bond coupling and an intranuclear tautomerization producing mycocyclosin (Figure 4) [15].

#### 2.4.2. C-H Hydroxylation and C-O Cleavage

**Figure 5:**
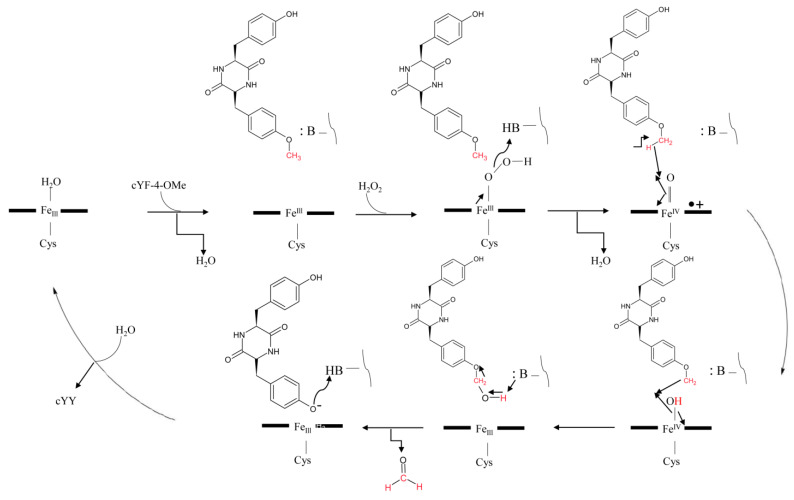
Schematic representation of the proposed mechanistic pathway of CYP121A1 hydroxylation and O-demethylation of cYF-4-OMe [9]. This figure is adapted from Ref. [9].

As indicated in the above section, it is clear that CYP121A1 functions like a peroxidase, and C-C intramolecular coupling proceeds via the formation of substrate radical intermediates. Also, as mentioned above, this type of reaction proceeds with the abstraction of a hydrogen atom from the hydroxyl group of the tyrosine moiety adjacent to the P450 heme. To probe this initial reaction step, a cyclodipeptide in the L-configuration, cyclo(L-Tyr-L-Phe-4-OMe) (cYF-4-OMe) was synthesized to remove the hydroxyl group on the aromatic ring and re-analyze the catalytic mechanism of CYP121A1 (Figure 5) [9].

The catalytic activity of CYP121A1 with cYF-4-OMe was tested through the peroxide shunt pathway using hydrogen peroxide or peracetic acid as the oxidant [9]. The results revealed a loss of a methyl group and production of cYY, indicative of an oxidative C-O bond cleavage reaction that is rarely observed for a P450 enzyme [9]. If CYP121A1 followed a peroxidase-like or classical P450-type response, the secondary product should be methanol or formaldehyde. Enzyme assays were performed using alcohol dehydrogenase (ADH) and formaldehyde dehydrogenase (FDH) to detect formaldehyde. Interestingly, formaldehyde was produced as opposed to methanol, suggesting that an *O*-demethylation reaction occurs instead of C-C bond coupling when cYF-4-OMe was used as a CYP121A1 substrate instead of cYY (Figure 5) [9].

The CYP121A1 reaction mechanism for the *O*-demethylation reaction proposed a compound I (Fe(IV)+•=O) species, which initiated a radical reaction with the methyl group via hydrogen atom abstraction [9]. The product of such a reaction was compound II (Fe(IV)-OH) and a radical on the carbon of the methyl group (Figure 5). The hydroxyl group from compound II reacts with the radical on the methyl group and forms the hydroxylated intermediate, cYF-4-OMeOH (Figure 5). Consequently, the hydroxylated product intermediate undergoes C-O bond cleavage via deformylation, producing the native substrate (cYY) and formaldehyde (Figure 5) [9]. The amount of cYY formed from the peroxide shunt pathway is not kinetically competent to outcompete the excess cYF-4-OMe, and, hence, mycocyclosin is not produced [9].

**Figure 6:**
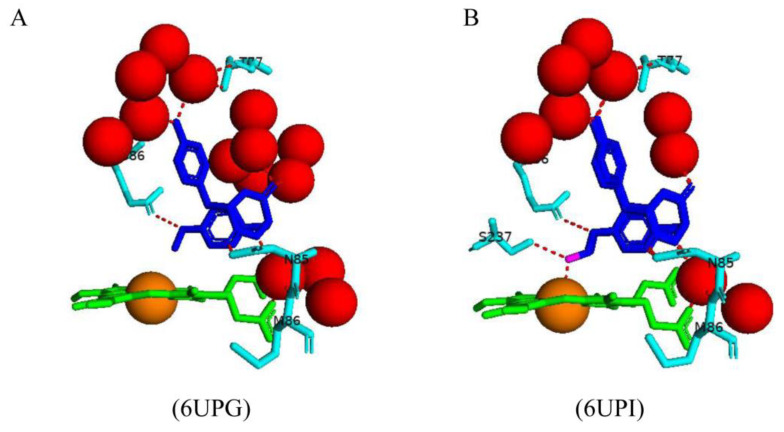
Analysis of CYP121A1 interactions with cyclo(L-Tyr-L-Phe-4-OMe) (cYF-4-Ome) (**A**) and its hydroxylated intermediate (**B**). Heme is shown in green, iron is shown as a brown sphere, ligand is in blue, and additional hydroxyl is shown in magenta. Amino acid residues sharing a direct and water-mediated polar interaction with the ligand are shown in cyan. Polar interactions are indicated as red dashed lines, water molecules are represented as red spheres, and amino acid residues are labeled according to their single-letter codes. PDB codes are shown within brackets underneath the respective model. A list of all amino acid residues within 5 Å of the ligands is shown in Table 2.

cYF-4-OMe binds in the distal pocket of CYP121A1 and is oriented with the methyl group pointing towards the heme [9] (Figure 6A). The ligand shared direct hydrogen bonds with five water molecules, Asn85 and Arg386 (Figure 6A). Thr77 and Met86 interacted with the ligand via water-mediated hydrogen bonds (Figure 6A). A hydroxylated intermediate was trapped and successfully crystallized in a complex with CYP121A1, revealing the hydroxylation site of this reaction [9]. This substrate intermediate was positioned in the same orientation as cYF-4-OMe and shared the same molecular interactions with Asn85, Arg386, Thr77, and Met86. The hydroxylated moiety of the intermediate shared a direct hydrogen bond with the sixth water ligand and Ser237 (Figure 6B). Similar to cYY, the sixth water ligand was not displaced, although it was involved in a hydrogen bond network.

These results conclusively prove that the hydrogen atom present in the hydroxyl group of the substrate plays a critical role in the C-C bond coupling reaction. CYP121A1 can switch from C-C coupling to an oxidative C-O bond cleavage via hydroxylation and then O-demethylation by insertion of an oxygen atom into cYF-4-OMe, resulting in the release of formaldehyde and cYY (Figure 5) [9]. These results indicate that the heme co-factor does not dictate the type of reaction catalyzed by CYP121A1 alone, as the substrate directs the oxidizing power to a specific chemical reaction.

### 2.5. CYP121A1 and Fluconazole Interactions Revealed a New Azole Drug–P450 Binding Mode

**Figure 7:**
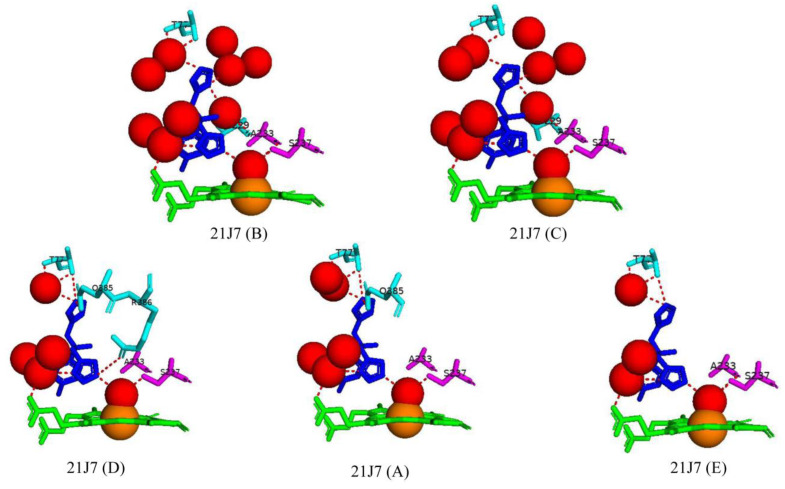
Analysis of the CYP121A1–fluconazole interactions concerning two I-helix residues, Ala233 and Ser237. The respective monomer name is shown in brackets next to the PDB code. Heme is shown in green, the iron atom is shown as a brown sphere, and the ligand (fluconazole) is shown in blue. Amino acid residues sharing a direct and water-mediated polar interaction with the substrate are shown in cyan. Manipulated I-helix residues are shown in magenta. Polar interactions are indicated as red dashed lines, water molecules are represented as red spheres, and amino acid residues are labeled according to their single-letter codes. A list of all amino acid residues within 5 Å of the ligand is shown in Table 2.

Azole drugs are known to interact with different P450, e.g., CYP51, such that the azole group substituents are perpendicular to the heme plane, and the azole nitrogen atom directly coordinates with the heme iron [24,25,26]. Structural analysis of CYP121A1 with bound fluconazole revealed a new azole drug–P450 binding mode whereby the fluconazole triazole, free nitrogen atom (N4) primarily coordinates the heme iron via a bridging water molecule and, to a lesser extent, directly to the iron (Figure 7) [18].

Unlike other P450s [24,25,26] where the I-helix seems to be flexible, the I-helix of CYP121A1 appears to be rigid, and the I-helix residues, Ala233 and Ser237, which are positioned above the heme plane, restricted the triazole group from occupying the ideal position for direct ligation [18]. Crystallization of CYP121 in a complex with fluconazole resulted in the formation of five monomers, namely, B, C, D, A, and E, where the alpha and beta carbon atom of Ala233 and the alpha carbon atom and hydroxyl group of Ser237 were shifted further away from the heme iron atom and resulted in the formation of direct contact of triazole nitrogen to the heme iron but far from the ideal positioning of fluconazole (Figure 7) [18].

Monomers B and C revealed fluconazole bound in one conformation, with one triazole nitrogen atom bound to the sixth water heme ligand and to an additional water molecule that was hydrogen-bonded to the heme propionate and two water molecules [18] (Figure 7). The hydroxyl group forms a water-mediated hydrogen bond with the oxygen of Thr229, and a second triazole nitrogen atom forms a direct hydrogen bond with one water molecule and water-mediated hydrogen bonds with Thr77, Thr229, and three water molecules (Figure 7). This conformation reveals that the sixth water heme ligand is not displaced from the heme iron. However, fluconazole occupies a position that allows for the formation of a direct hydrogen bond to the sixth water heme ligand (Figure 7) [18].

Interestingly, monomers D, A, and E revealed a second molecular conformation whereby the triazole moiety of fluconazole could ligate directly with the heme iron. However, the triazole nitrogen atom is slightly shifted away from the iron atom, and the triazole group does not bind perpendicularly to the heme [18]. The close contacts between the triazole group and CYP121A1 I-helix residues Ala233 and Ser237 could explain this finding. Fluconazole revealed a similar interaction in monomer D compared to monomers B and C. However, the triazole group that interacts with the sixth water heme ligand forms a direct hydrogen bond with Arg386, and a second triazole group forms a water-mediated hydrogen bond with Thr77 and a direct hydrogen bond with Gln385 (Figure 7). Fluconazole interacted with fewer water molecules than monomers B and C, which the interaction of Gln385 and Arg386 could cause due to the I-helix moving further away and relieving some constraints.

Monomer A followed a similar pattern to monomer D with slight changes; the triazole group interacting with Thr77 also interacted with an additional water molecule, and the direct hydrogen bond with Arg386 was absent, which could be due to Ala233’s position (Figure 7). The distance between the I-helix residues and the heme iron was the largest in monomer E [18]. In this monomer, the triazole group had a direct hydrogen bond to the sixth water heme ligand and two additional water molecules, and a second triazole group had a water-mediated hydrogen bond with Thr77 (Figure 7). Compared to the other monomers, fluconazole interacted with the fewest water molecules and only interacted with one amino acid residue. The shifting of the I-helix greatly impacted the position of the ligand and, thus, its interactions with amino acid residues. This further highlights the importance of the I-helix position in determining the azole binding mode.

In all CYP121A1 monomers, fluconazole is anchored in the active site by hydrophobic interactions with the amino acids Met62, Val78, Val82, Val83, and Phe168 [18] (Table 2). Fluconazole occupied 30% of the direct ligation mode in monomer D and 50% in A and E, indicating further that the I-helix residues were shifted away from the heme iron the closer fluconazole moved towards the ideal position for direct ligation as the constraints surrounding the heme environment was lessened [18]. The CYP121A1-fluconazole structure demonstrates for the first time that azole coordination of P450 heme iron can occur via a water molecule directly to the iron atom.

### 2.6. The Presence of the DKP Ring, Tyrosyl Side Chains, and the Substrate’s Configuration Impact the Enzymatic Activity of CYP121A1

To further understand the substrate and catalytic specificities of CYP121A1, 14 analogs of the substrate cYY were synthesized and bound to CYP121A1, of which five were successfully crystallized in complex with the protein. Specifically, the tyrosyl side chains or the DKP ring were modified [27].

**Figure 8:**
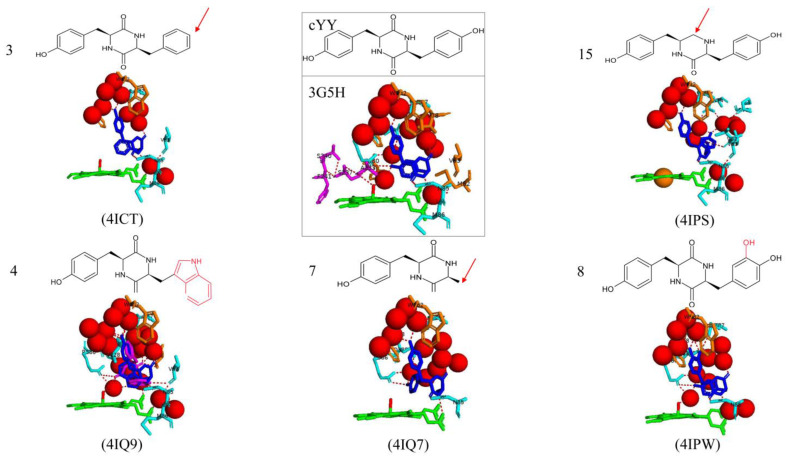
Analysis of CYP121A1 interactions with native and five substrate cyclo(L-Tyr-L-Tyr) (cYY) mutants. Red arrows indicate omitted parts of cYY; additional parts to cYY mutants are outlined in red. Native substrate and its interactions with CYP121A1 structure are shown within the box. Heme is shown in green, the sixth water ligand is shown as a red stick, iron is shown as a brown sphere, ligand one is shown in blue, and ligand two is shown in magenta where applicable. Amino acid residues sharing a direct and water-mediated polar interaction with the ligand are shown in cyan, and amino acid residues sharing van der Waal interactions are shown in orange. Polar interactions are indicated as red dashed lines, water molecules are represented as red spheres, and amino acid residues are labeled according to their single-letter codes. PDB codes are within brackets, underneath the respective model. A list of all amino acid residues within 5 Å of the ligands is listed in Table 2.

To evaluate the role of the chirality of tyrosine residues in cYY, cyclo(L-Tyr-D-Tyr) and cyclo(D-Tyr-D-Tyr) were tested with CYP121A1 [27]. They included analogs 3, 4, and 7 (Figure 8). Analog 8 is a cyclodipeptide that has one additional hydroxyl group located on the specific carbon atom involved in the C–C coupling catalyzed by CYP121A1, and analog 15 differs from cYY by the reduction of one of the keto moieties of the DKP ring to CH_2_ [27] (Figure 8). All substrate analogs showed similar contacts with CYP121A1 active site residues, namely, Asn85, Phe168, and Trp182.

The reduction of one keto moiety of the DKP ring in analog 15 significantly impaired binding to CYP121A1, and spectrophotometric studies revealed a different binding type [27]. Very little or no binding was observed for analog seven, which comprised one aliphatic side chain. However, compounds with two aromatic side chains showed significant binding to CYP121A1. These included analogs 3, 4, and 8 (Figure 8). These three compounds showed Type I substrate-like binding with low-to-high spin transition of the heme iron [27]. Interestingly, no evidence for the binding of cyclo(L-Tyr-D-Tyr) and cyclo(D-Tyr-D-Tyr) to CYP121A1 was observed in both titration and inhibition studies [27]. This finding indicates the importance of the aromatic side chains and their stereochemistry regarding the CYP121A1 catalytic mechanism.

The enzymatic transformation of these compounds by CYP121A1 was investigated, and studies revealed that only compound 4 was significantly metabolized (50%) in activity assays. However, more than ten different metabolite products were detected, revealing that the reaction was non-specific [27]. Due to the absence of binding of analog 7, no metabolites were observed after incubation [27]. Inefficient conversion was observed for analog 15; 98% of the compound remained after incubation [27]. Therefore, no cYY mutants were selectively or efficiently transformed by CYP121A1, indicating a high specificity for cYY.

All analogs bound to the active site of CYP121A1 in a similar orientation found for cYY (Figure 8), indicating a standard binding mode. The sixth water ligand was not displaced for all mutants as observed in cYY binding, and van der Waal interactions between Phe168 and Trp182 that were present upon cYY binding were observed for all substrate analogs [27] (Figure 8). The direct hydrogen bond formed between a carbonyl group of the DKP ring and the nitrogen atom of Asn85 was present for all analogs except for the second conformation observed for analog 4 (Figure 8).

The active site orientation of analog 3 was very similar to cYY, except for less water-mediated bonds with water molecules and a hydrogen bond with Val83 (Figure 8). The significant difference between the CYP121A1 binding of analog 3 and cYY was due to the absence of the hydrogen bond between a water molecule that connected the substrate to the sixth water ligand, creating the hydrogen bond network [27] (Figure 8). This finding highlights the importance of the missing hydroxyl group for establishing the hydrogen bond network above the CYP121A1 heme.

Two alternate conformations were observed at the same CYP121A1 binding site for analog 4 (Figure 8). In conformation, 1 was very similar to cYY; the tyrosyl side chains pointed toward helices F and G, and the tryptophan moiety faces the DKP ring and is located above the heme. A nitrogen atom of the tryptophanyl group interacts with the iron atom via one water molecule, and it shares a water-mediated hydrogen bond with Thr77, Met86, and Val228 (Figure 8). In conformation 2, the tyrosyl side chains occupy roughly the same position. Still, the tryptophan moiety and DKP ring interchange, and therefore, the DKP ring is located above the heme and interacts with the heme iron via a water molecule [27]. The direct hydrogen bond between the substrate and Asn85 is replaced with an ionic bond between a nitrogen atom of the tryptophan moiety and the carbonyl group of Val83 (Figure 8). Both conformations interact with the heme propionate groups.

In the structure of analog 7, the tyrosyl side chain faces the DKP ring. The hydroxyl group is positioned just above the heme and forms a direct hydrogen bond with one nitrogen atom of Arg386, which interacts with Gln385 through one water molecule (Figure 8). In the crystal structure of substrate analog 8, the ligand occupies the same position as cYY. Mutant 8 contains an additional hydroxyl group, which is involved in two direct hydrogen bonds with the oxygen atom of Thr77 and the oxygen atom of the carbonyl of Ala167 (Figure 8). Analog 15 binds to CYP121A1 in a similar manner to the other ligands (Figure 8). The primary difference involves the second tyrosyl that does not face the DKP ring; therefore, the hydrogen bonding network previously observed is largely disrupted [27].

Various interactions between the ligands and specific CYP121A1 residues were required for efficient binding, namely, the hydrogen bond of Asn85 and van der Waal interactions between Phe168 and Trp182. The structure of the DKP ring was a key feature of the ligand binding as the reduction of one of its keto moieties substantially reduced binding to the CYP121A1 protein [27]. The absence or incorrect position of the hydroxyl group led to the disruption of the hydrogen bond network above the heme. This resulted in the impairment of the low-to-high spin transition of the heme iron, highlighting the importance of the presence and correct location of the hydroxyl group for efficient transformation.

### 2.7. Analogs of cYY with Methyl and Halogen Groups Reveal the CYP121A1 Substrate Specificity

As indicated in Section 2.6, CYP121A1 is highly substrate-specific, and the conservation of the DKP ring is essential for catalytic function [14,27]. To investigate the role of the tyrosyl phenolic groups on the binding affinity of CYP121A1 further, various analogs of cYY were designed, and nine were successfully crystallized in complex with the P450 protein.

The phenolic group was the main target for mutagenesis and was changed in various ways, including the removal of one or both phenolic groups, one or both phenolic groups blocked with the addition of O-methyl groups, and the addition of halogens or methyl groups to the phenols on the tyrosyl rings [28]. These additions were either at an ortho- or meta-position to investigate the steric and electronic effects on binding to CYP121A1.

UV–visible difference spectroscopy was used to analyze the binding affinities of all mutants. Interestingly, iodinated compound 11 and O-Me analog 5 resulted in a complete shift from a low to a high spin state indicative of tight binding with the displacement of the sixth water ligand [28]. The addition of one methylated phenolic group in analog 5 resulted in an increase in the binding affinity compared to the native substrate (KD = 10.4 μM) relative to cYY (KD = 30 μM) [28]. The same pattern was noticed with analogs containing halogen substituents at position 3 of the phenol rings (analogs 11–14). The binding affinity increased with an increase in the size of the halogen; F < Cl < Br < I [28].

Introducing a methyl group at the third position increased binding affinity constants (analog 15) compared to cYY; however, it was not larger than analog 11 [28]. Introducing a second methyl group from the 3,5-dimethyl analog (mutant 16) reduced the binding affinity. Analog 17 had an additional methyl group at the 2-position, displaying slightly decreased binding affinity relative to cYY. The 2,6-dimethyl analog, however, exhibited no binding to CYP121A1 [28]. Thus, the position of the methyl group on the phenol ring greatly impacted the binding affinity to CYP121A1.

**Figure 9:**
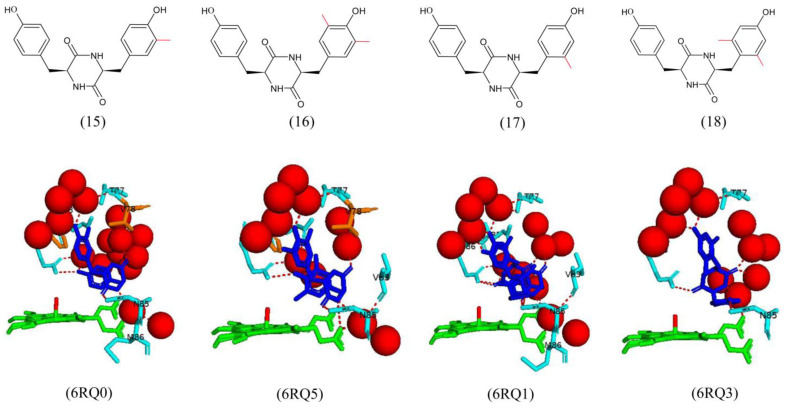
Analysis of CYP121A1 interactions with four methylated analogs of cyclo(L-Tyr-L-Tyr) (cYY). The methyl groups added to cYY analogs are highlighted in red in the chemical structures shown in the upper panel. Heme is shown in green, the sixth water ligand is shown as a red stick, and the ligand is shown in blue. Amino acid residues sharing a direct and water-mediated polar interaction with the ligand are shown in cyan, and amino acid residues sharing a hydrophobic interaction with the ligand are shown in orange. Polar interactions are indicated as red dashed lines, water molecules are represented as red spheres, and amino acid residues are labeled according to their single-letter codes. PDB code and mutant numbers are shown within brackets underneath the respective model and chemical structure. A list of all amino acid residues within 5 Å of the ligands is shown in Table 2.

Analog 15 binds efficiently in the CYP121A1 binding pocket of the enzyme active site. The binding of the ligand does not displace the sixth water ligand [28]. Mutant 15 shares a direct hydrogen bond between the carbonyl group of the DKP ring and the side chain of Asn85 (Figure 9). The ligand also shares water-mediated hydrogen bonds between the phenolic groups and Thr77, Met 86, Gln385, and Arg386. Hydrophobic interactions between the ligand and Phe168 and Val78 are present [28] (Figure 9). Mutant 16 binds similarly to mutant 15, but the 3,5-dimethyl substituted aromatic ring was oriented towards the heme. All hydrogen bonds, water-mediated bonds, and hydrophobic interactions with amino acid residues are the same as observed for mutant 15, except for a water-mediated hydrogen bond with Val83 and the absence of the water-mediated bond with Met86 (Figure 9). Both mutants interact with the heme propionate group. Mutant 17 binds in a similar manner to mutant 16 with the substituted aromatic ring located in the pocket near the heme and interacts with the heme propionate group via a water molecule [28] (Figure 9).

Analog 18 binds in the enzyme active site in a flipped conformation compared to analogs 15 and 16 (Figure 9). The aromatic ring containing methyl substituents at positions 2 and 6 binds in the P450 distal pocket, oriented away from the heme moiety. Additionally, the unsubstituted phenolic group is situated in the space occupied by the DKP ring of the natural substrate, cYY [28]. The non-methylated aromatic ring shares a direct hydrogen bond with Asn85, the DKP ring shares a direct hydrogen bond with Arg386, and an additional hydrogen bond is observed between the nitrogen atom of the DKP ring and a water molecule (Figure 9). The water-mediated hydrogen-bonding interactions between the carbonyl groups on the DKP ring and Thr77 are still present. However, the water-mediated interaction with Gln385 is absent. The ligand interacts with the heme propionate via a water molecule (Figure 9).

**Figure 10:**
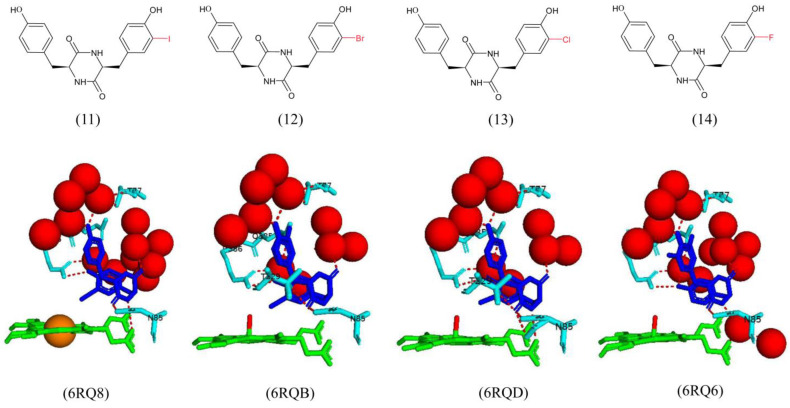
Analysis of CYP121A1 interactions with four halogen analogs of cyclo(L-Tyr-L-Tyr) (cYY). The halogen groups added to cYY analogs are highlighted in red in the chemical structures shown in the upper panel. Heme is shown in green, the sixth water ligand is shown as a red stick, iron is shown as brown, and the ligand is shown in blue. Amino acid residues sharing a direct and water-mediated polar interaction with the ligand are shown in cyan. Polar interactions are indicated as red dashed lines, water molecules are represented as red spheres, and amino acid residues are labeled according to their single-letter codes. PDB code and mutant numbers are shown within brackets underneath the respective model and chemical structure. A list of all amino acid residues within 5 Å of the ligands is shown in Table 2.

Regarding the halogen substituents, analog 14 binds in a similar fashion as the natural substrate. However, the substituted aromatic ring binds in the distal pocket [28]. The ligand is bound in a similar orientation and shares the same hydrogen bond interactions with amino acid residues as analog 15 (Figure 10). Water-mediated hydrogen bonds are present between the ligand and Thr77 and Gln385 to the heme propionate group (Figure 10). Analogs 12 and 13 bind in a similar manner to the natural substrate, but in contrast to analog 14, the halogen-substituted aromatic ring binds in the heme pocket [28]. The binding of these ligands did not displace the sixth water ligand. Both analogs share a direct hydrogen bond with Asn85, Thr229, and Arg386 and water-mediated hydrogen bonds with Thr77 and Gln385 (Figure 10).

**Figure 11:**
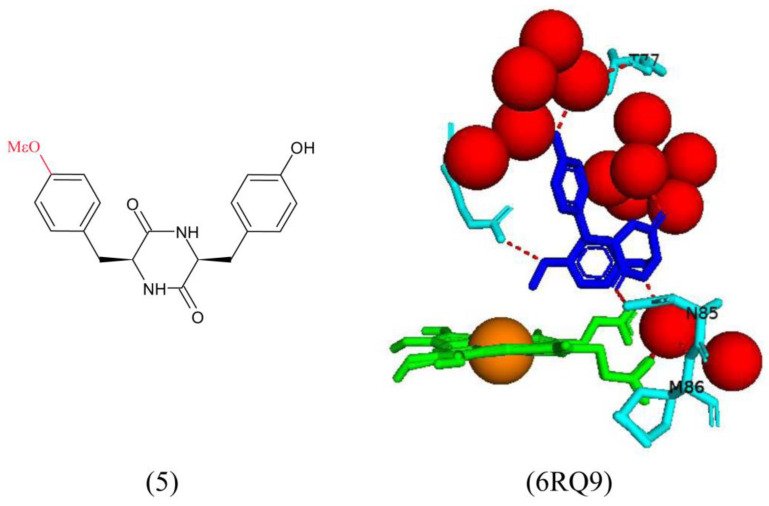
Analysis of CYP121A1 interactions with an O-methyl group analog of cyclo(L-Tyr-L-Tyr) (cYY). The O-methyl group added to the cYY analog is highlighted in red in the chemical structure shown in the left panel. Heme is shown in green; iron is shown as a brown sphere, and ligand is shown in blue. Amino acid residues sharing a direct and water-mediated polar interaction with the ligand are shown in cyan. Polar interactions are indicated as red dashed lines, water molecules are represented as red spheres, and amino acid residues are labeled according to their single-letter codes. PDB code and mutant number are shown within brackets, underneath the respective model and chemical structure. A list of all amino acid residues within 5 Å of the ligand is shown in Table 2.

For the methyl analogs, the substituted aromatic ring binds in the P450 proximal pocket close to the heme for analog 5 (Figure 11). The sixth water ligand is completely displaced, which correlates with the large high-spin signal observed in both the EPR and UV–visible spectra [28]. The aromatic side chain is positioned; so, the OMe group is directly above the heme iron atom (Figure 11). The high binding affinity of this analog suggests specific interactions between the methyl group and the iron atom [28]. The OMe moiety shares a direct hydrogen bond with Arg386, and the DKP ring shares a direct hydrogen bond with Asn85. The ligand also shares water-mediated bonds with Thr77 and Met86 and the heme propionate (Figure 11). Analog 11 also binds with the substituted aromatic ring located in the proximal pocket and the sixth water ligand is completely displaced. The iodine atom is situated very close to the heme iron atom, confirmed by the large high-spin signal observed in both the EPR and UV–visible spectra [28]. Water-mediated hydrogen bonds are present between the ligand and Thr77 and Gln385 (Figure 11). The DKP ring shares a direct hydrogen bond with Asn85, and the phenolic group on the substituted aromatic ring shares a direct hydrogen bond with Arg386, indicating tight binding and correlating with the high binding affinity observed for this compound (KD = 0.28 μM).

These results suggest that the phenolic groups of the native substrate are not essential for binding as substrate analogs with one or both of the phenol groups removed, or methylated, or replaced with iodine atoms showed similar or increased binding affinity [28]. Analog 5 had an increased binding affinity (KD = 10.4 μM) and induced a shift from low-to-high spin for the heme iron atom. This may be due to the favorable interactions of the CH_3_ group and the heme iron. The aryl-methylated analogs (15–18) revealed critical information about the steric effects of substrate binding to CYP121A1. Substitutions at the 2-position of the aromatic ring were unfavorable. However, the introduction of a single methyl group at this position on the ring results in only a slight decrease in binding affinity [28]. This analog bound in a flipped conformation; thus, unfavorable interactions between the methyl group and the heme are avoided. Analog 15 has a methyl group at the 3-position and revealed an increased binding affinity (KD = 1.1 μM), possibly due to increased hydrophobic interactions between the methyl moiety and Phe168 and Val78 [28].

Regarding the halogen substrate analogs (11–14), iodine is larger in size and polarizability. It thus binds with favorable molecular interactions between the halogen and the heme group, resulting in a higher binding affinity. Bromine and chlorine have a decreased size and polarizability, resulting in a poorer affinity and a lack of displacement of the sixth water ligand [28]. The small, non-polarizable fluorine may have resulted in insignificant steric clashes with nearby amino acid residues, with minor increases in hydrophobic interactions, leading to increased binding affinity. Despite the different binding affinities of each separate analogs, CYP121A1 could not turnover these analogs, and all compounds revealed little or no antimicrobial activity [28]. The authors suggested that the dense, waxy, lipid-rich cell wall of *M. tuberculosis* was a major barrier to the penetration of these compounds into the bacterial cell. However, critical information was revealed on the steric effects and interactions between the ligands and CYP121A1 active site residues.

### 2.8. Fragment-Based Chemical Library Approaches Design Potential Potent CYP121A1 Inhibitors

Based on the available information regarding the substrate specificity and catalytic mechanism of CYP121A1, attempts have been made to design potent inhibitors against this P450. Typical P450 inhibitors such as fluconazole and iodopyrazole, despite binding to CYP121A1, did not successfully inhibit this enzyme [14,18]. Azole drugs may bind to CYP121A1 but have low oral bioavailability or cause major toxic side effects due to their broad-spectrum inhibitory activity against human P450s [29]. Therefore, there is growing interest in developing more potent and selective inhibitors of *M. tuberculosis* P450s.

Fragment-based approaches represent a new technique that involves the development of small-molecule ligands as chemical tools and leads to drug development [30]. This effective technique uses structural guidance to design and synthesize potent ligands from low-molecular-weight, weaker-binding fragment molecules [30]. This method has been applied to various molecular targets to identify new inhibitory molecules [29]. Here, we overview the different approaches to developing CYP121A1 inhibitory molecules that resulted in further understanding of CYP121A1 interactions with other molecules.

#### 2.8.1. Designing Inhibitors That Coordinate with the Heme Iron

**Figure 12:**
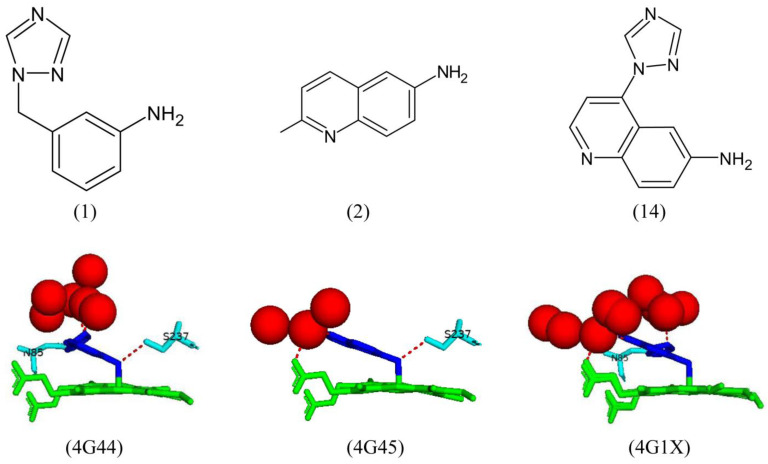
Analysis of CYP121A1 interactions with two parent fragments (1 and 2) and the merged fragment 14. The chemical structures of the compounds are shown in the upper panel, and their interactions with CYP121A1 are shown in the lower panel. Heme is shown in green, and ligands are shown in blue. Amino acid residues sharing a direct and water-mediated polar interaction with the ligands are shown in cyan. Polar interactions are indicated as red dashed lines, water molecules are represented as red spheres, and amino acid residues are labeled according to their single-letter codes. PDB code and fragment number are shown within brackets underneath the respective model and chemical structure. A list of all amino acid residues within 5 Å of the ligands is shown in Table 2.

Fragment-based screening with CYP121A1 revealed 26 molecules that may bind within the active site and had potential interactions with CYP121A1. Eight of these fragments had the highest solubility and thus were successfully crystallized in complex with the enzyme [30]. Fragments 1–4 bound within the distal pocket of the P450 active site, and no significant conformational change was observed [30]. Fragments 1 and 2 coordinate with the heme iron atom via an aryl-amine nitrogen, which occupies the sixth water ligand position (Figure 12). Both fragment amine groups share a direct hydrogen bond with Ser237. The 1,2,4-triazole moiety of fragment 1 shares a direct hydrogen bond with Asn85, and the quinoline nitrogen of fragment 2 shares a direct hydrogen bond with the heme carboxylate group [30] (Figure 12). The aromatic moieties of both fragments are oriented in a near parallel position to the P450 heme. Fragment 14 also coordinates the heme iron and shares a direct hydrogen bond with Asn85 via its 1,2,4-triazole moiety, similar to fragment 1. It also interacts with the heme carboxylate group, similar to fragment 2 [30]. This combination of interactions was to be expected as fragment 14 was designed by merging both fragments 1 and 2 together (Figure 12).

**Figure 13:**
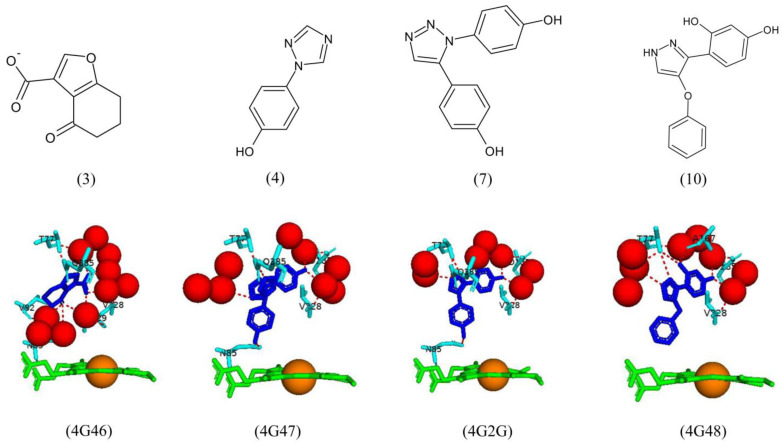
Analysis of CYP121A1 interactions with two parents (3 and 4), a biaryl-substituted azole analog of fragment 4, and one merged fragment (10). The chemical structures of the compounds are shown in the upper panel, and their interactions with CYP121A1 are shown in the lower panel. Heme is shown in green, iron is shown as a brown sphere, and ligands are shown in blue. Amino acid residues sharing a direct and water-mediated polar interaction with the ligands are shown in cyan. Polar interactions are indicated as red dashed lines, water molecules are represented as red spheres, and amino acid residues are labeled according to their single-letter codes. PDB code and fragment number are shown within brackets underneath the respective model and chemical structure. A list of all amino acid residues within 5 Å of the ligands is shown in Table 2.

Fragments 3 and 4 are located further from the heme iron. Fragment 3 shares a direct hydrogen bond with Thr77, Gln385, and six water molecules (Figure 13). Water-mediated hydrogen bonds are shared between fragment 3 and Asn85, Val82, Val28, and Thr229 (Figure 13). Fragment 4 forms a direct hydrogen bond with three water molecules, its phenol group with Asn85, and its triazole nitrogen atoms formed hydrogen bonds with Thr77 and Gln385 (Figure 13).

Fragments 3 and 4 were merged to form fragment 10, which oriented similarly and shared similar hydrogen bond interactions with active site residues as well as a new hydrogen bond with Ala167 and water-mediated bonds with Asp185 and Val228 (Figure 13). Interestingly, the direct hydrogen bonds with Asn85 and Gln385 were not present. Fragment 7 was a biaryl-substituted azole analog of fragment 4 and was oriented similarly. It shared all direct and water-mediated hydrogen bonds as fragment 4, besides one fewer water molecule (Figure 13). These results, along with ligand binding assays, reveal fragment 14 as a leading drug candidate as it has the highest ligand binding efficiency and is coordinated with the heme iron, which may ultimately disrupt the catalytic mechanism of CYP121A1, leading to cell death [30].

#### 2.8.2. Substitutions of Aromatic Rings Revealed Binding Hotspots within the CYP121A1 Active Site

**Figure 14:**
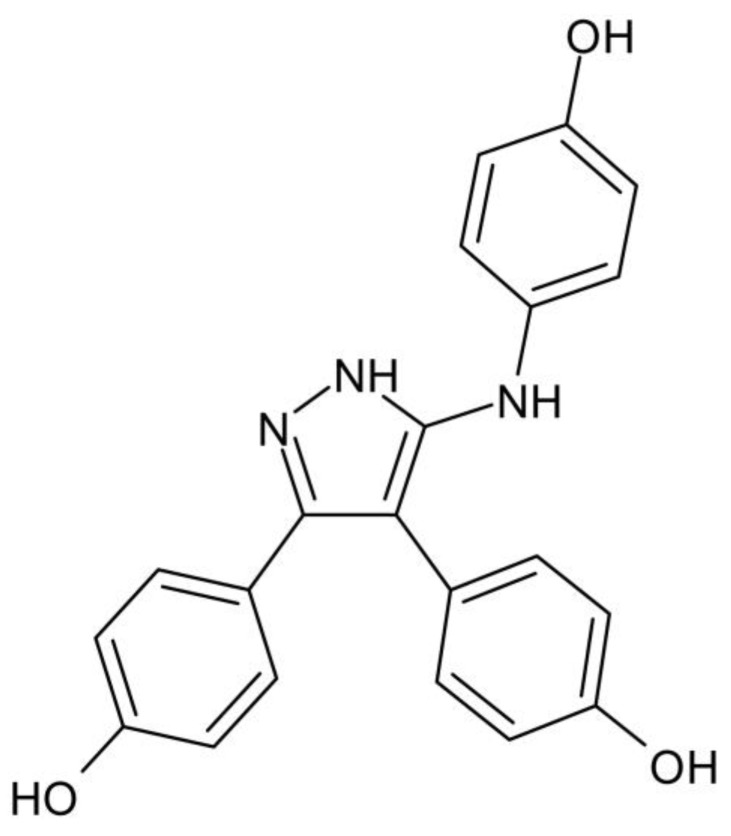
Chemical structure of a triphenol pyrazole-amine compound known to interact with CYP121A1.

Fragment-based studies revealed a triphenyl pyrazole-amine compound (Figure 14) that displayed optimized hydrogen bonding interactions between the 5-amino pyrazole core and distal active site residues Gln385, Ala167, and Thr77 of CYP121A1 [29]. This hydrogen bonding interaction represented a novel binding mode for an azole-containing compound, which usually binds directly to the heme iron through a heterocyclic nitrogen atom. These interactions were thus referred to as possible binding hotspots. However, this compound had a low ligand binding efficiency and was deconstructed into its relative parts [29]. Retro-fragmentation of this compound into its components was used to assess the binding contribution of the three aromatic rings surrounding the amino-pyrazole core [29] (Figure 14).

**Figure 15:**
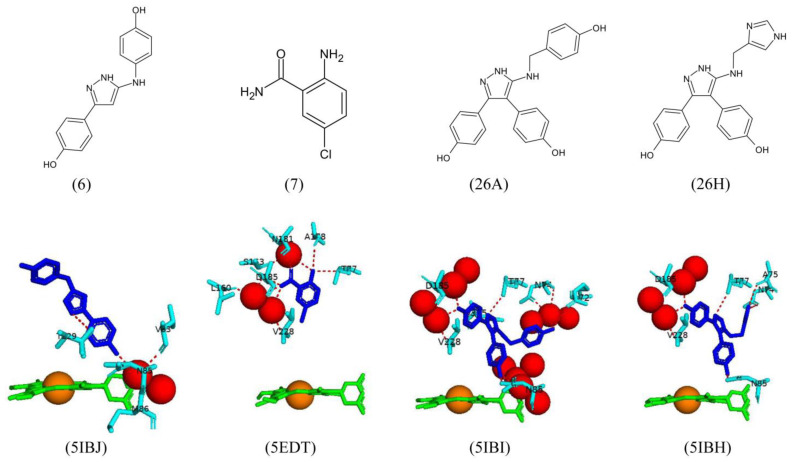
Analysis of CYP121A1 interactions with triphenol pyrazole-amine fragment molecules. The chemical structures of the compounds are shown in the upper panel, and their interactions with CYP121A1 are shown in the lower panel. Heme is shown in green, iron is shown as a brown sphere, and ligands are shown in blue. Amino acid residues sharing a direct and water-mediated polar interaction with the ligands are shown in cyan. Polar interactions are indicated as red dashed lines, water molecules are represented as red spheres, and amino acid residues are labeled according to their single-letter codes. PDB code and fragment number are shown within brackets underneath the respective model and chemical structure. A list of all amino acid residues within 5 Å of the ligands is shown in Table 2.

Fragment 6 was designed by removing one aromatic ring from the compound. This compound was bound to CYP121A1 in a flipped conformation (Figure 15). In this orientation, the pyrazole moiety shared direct hydrogen bonds with Thr229, and the hydroxyl group closest to the heme iron shared water-mediated bonds with Met86, Asp85, and Val83 (Figure 15). Removing one aromatic ring did not affect the binding of fragment 6. However, the hydrogen bond interactions differed from the triphenyl pyrazole-amine compound [29]. A substituted benzamide fragment, fragment 7, was designed to test the effects on polar interactions between Thr77 and Asp185 [29]. Despite the substitutions, the fragment formed direct hydrogen bonds with Thr77, Ala178, and Asp185 and water-mediated bonds with Ser163, Asn181, and Val228 (Figure 15). The substitution of chlorine into the compound and changing the position of the aromatic ring did not affect the hydrogen bonding interactions, thus indicating that these changes will unlikely cause any conformational strain [29].

Fragments 26H and 26A were designed by mutating one aromatic ring only. Fragment 26A formed direct hydrogen bonds with Asn85, Arg72, Thr77, Asn74, and Gln385, as well as water-mediated bonds with Asp185 and Val228 (Figure 15). Fragment 26H was bound within the binding pocket with the imidazole ring binding distal to the heme cofactor and occupying the space of the missing aromatic ring [29] (Figure 15). This fragment formed direct hydrogen bonds with Asn85, Asn74, Ala75, and Thr77 and water-mediated bonds with Asp185 and Val228 (Figure 15). Hydrogen bonding interactions with Asn74 and Ala75 had not been previously observed in other CYP121A1 crystal structures to date and could provide a new route for designing potential inhibitors. Despite these novel interactions, the binding affinity of 26H was weak and could not be considered a potential inhibitor [29].

**Figure 16:**
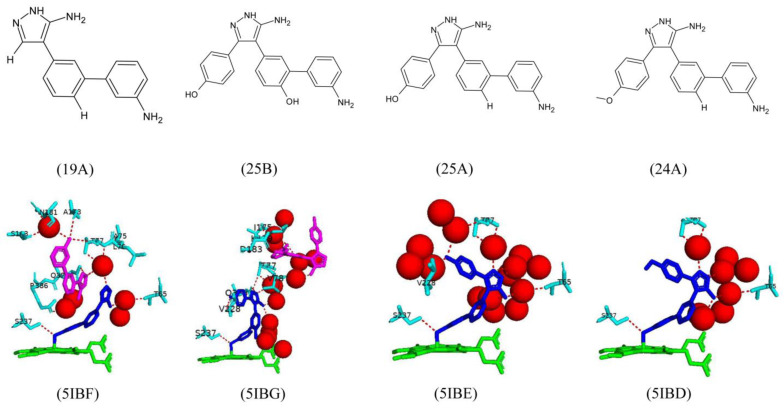
Analysis of CYP121A1 interactions with triphenol pyrazole-amine fragment molecules with substitutions at the aromatic rings. The chemical structures of the compounds are shown in the upper panel, and their interactions with CYP121A1 are shown in the lower panel. Heme is shown in green, iron is shown as a brown sphere, and ligands are shown in blue and magenta where applicable. Amino acid residues sharing a direct and water-mediated polar interaction with the ligands are shown in cyan. Polar interactions are indicated as red dashed lines, water molecules are represented as red spheres, and amino acid residues are labeled according to their single-letter codes. PDB code and fragment number are shown within brackets underneath the respective model and chemical structure. A list of all amino acid residues within 5 Å of the ligands is shown in Table 2.

Fragment 19A was designed by removing two aromatic rings and adding aromatic rings to the existing ones; however, it had a nitrogen-containing ring attached. This compound was bound in two different conformations (Figure 16). One conformation interacts directly with the heme iron and forms a direct hydrogen bond with Ser237 and water-mediated bonds with Thr65, Thr65, and Thr77 (Figure 16). The second conformation is bound further away from the heme and forms direct hydrogen bonds with Thr77, Ala178, and Gln385 (Figure 16). This compound had a higher binding affinity than the original fragment; however, fragment 25B was structurally similar to fragment 19A, besides the aromatic ring, which was reincorporated into the phenol ring and surprisingly had an even higher binding affinity [29].

Fragment 25B was also bound in two different conformations, with one conformation interacting directly with the heme iron and the other further in the binding pocket (Figure 16). The first conformation interacted and formed direct hydrogen bonds with Ser237, Val228, Gln385, and Thr77, whereas the second conformation only had one direct hydrogen bond with Asp183 and water-mediated bonds with Lys179, Val78, and Ile175 (Figure 16). This compound matched all binding hotspots, and the 3-amino group shared a hydrogen bond with Ser237, which is known to be the conserved alcohol residue of CYP121A1 required for the catalysis of proton transfer to the heme iron and the organization of the sixth water ligand [29].

Removal of the hydroxyl group on one of the aromatic rings led to fragment 25A and the design of 24A. Fragment 25A directly coordinated the heme iron but was oriented at a different angle than 25B [29]. This fragment formed a direct hydrogen bond with Ser237 only and water-mediated bonds with Thr77, Thr65, and Val228 (Figure 16). Removing the 4-hydroxyl group may have optimized the stacking interactions between the ligand and active site residues. Fragment 24A is coordinated with the heme iron. It is oriented identically to fragment 25A and shares the same hydrogen bond interactions with fragment 25A, besides the lack of the water-mediated bond with Val228 due to the negatively charged oxygen atom on the aromatic ring (Figure 16). Despite all substitutions and interactions, no fragments inhibited bacterial growth significantly. However, this study highlighted the essential binding hotspots within the active site on CYP121A1 and the critical interactions between aromatic rings and active site residues [29]. This will allow for a second generation of new inhibitors for evaluation.

### 2.9. Y-Shaped Imidazole and Lipophilic Pyrazole Derivatives Potentially Bind and Block the Substrate Access Channel of CYP121A1

The endogenous substrate of CYP121A1 can be considered “Y-shaped.” Therefore, biaryl pyrazole, imidazole, and triazole derivatives were designed in the same molecular shape to assess their effect on CYP121A1 ligand binding, as well as their antimicrobial activity. Only two imidazole derivatives were successfully crystallized in complex with the enzyme (7B and 7E) [31].

**Figure 17:**
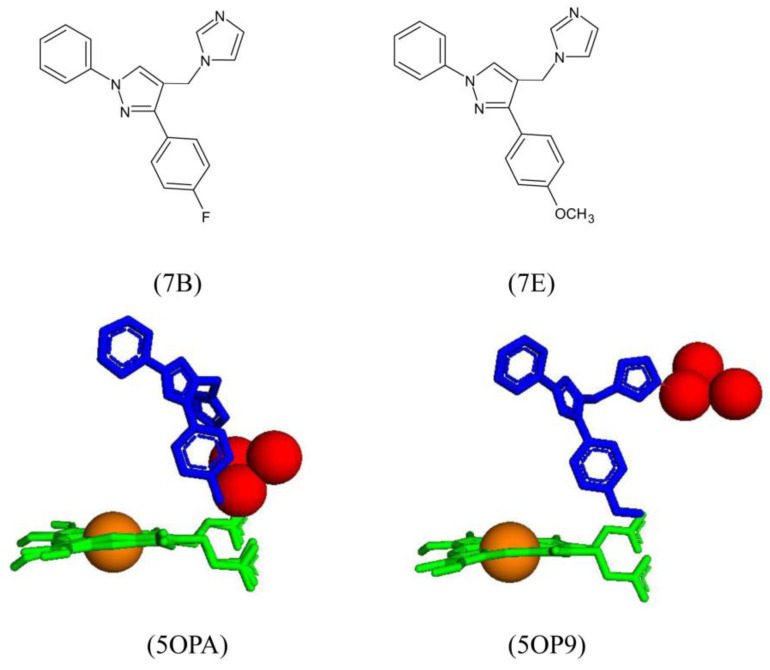
Analysis of CYP121A1 interactions with two aryl-substituted imidazole compounds. The chemical structures of the compounds are shown in the upper panel, and their interactions with CYP121A1 are shown in the lower panel. Heme is shown in green, iron is shown as a brown sphere, and ligands are shown in blue. Water molecules are represented as red spheres. PDB codes and derivative numbers are shown within brackets underneath the respective model and chemical structure. A list of all amino acid residues within 5 Å of the ligands is shown in Table 2.

The ligand binding affinity results suggest that the ligand binding mode could involve the imidazole and the nitrogen moiety interacting with the heme iron via the sixth water ligand [31]. However, the crystallized structures of two derivatives reveal that the imidazole moiety binds too far away from the heme iron for direct or indirect interaction. Interestingly, neither derivative interacts with amino acid residues, and these only share a direct hydrogen bond with one water molecule (Figure 17). However, both derivatives were oriented similarly and could restrict the substrate access channel, inhibiting CYP121A1 by blocking substrate access [31] (Figure 17). This finding correlates with the anti-microbial activity results that showed imidazole derivatives were generally more inhibitory to *M. tuberculosis* growth, displayed better activity, and were significantly more active than the triazole drugs, e.g., fluconazole [31].

These results reveal the importance of the shape of the ligand within the active site and how it influences the binding affinity and anti-microbial activity of potential inhibitors. The binding location of these ligands and the anti-microbial data suggest that a Y-shaped inhibitor could block the substrate access channel and thus lead to the inhibition of CYP121A1.

Diphenyl pyrazole derivatives bind the active site of CYP121A1 similarly to fluconazole and cYY [10,18]. These compounds all interact with the P450 heme via a water molecule. To potentially increase the binding affinity and interaction with the heme, the carbon chain between the pyrazole entity and the heme was extended [32]. Furthermore, to investigate the relationship between lipophilicity and antimycobacterial activity, pyrazole rings were substituted with alkyl and biaryl groups [32].

Biaryl derivatives induced the most extensive CYP121A1 Soret absorbance shifts, similar to azole drugs such as fluconazole and ketoconazole, compounds known to bind tightly to CYP121A1 [18,32]. Interestingly, the lipophilic compounds showed optimal antimycobacterial activity. The binding affinity of the extended pyrazole derivatives (KD = 3.31 μM) was higher than that of other compounds due to this derivative occupying a binding site similar to that of the natural substrate cYY and the inhibitor fluconazole [32].

**Figure 18:**
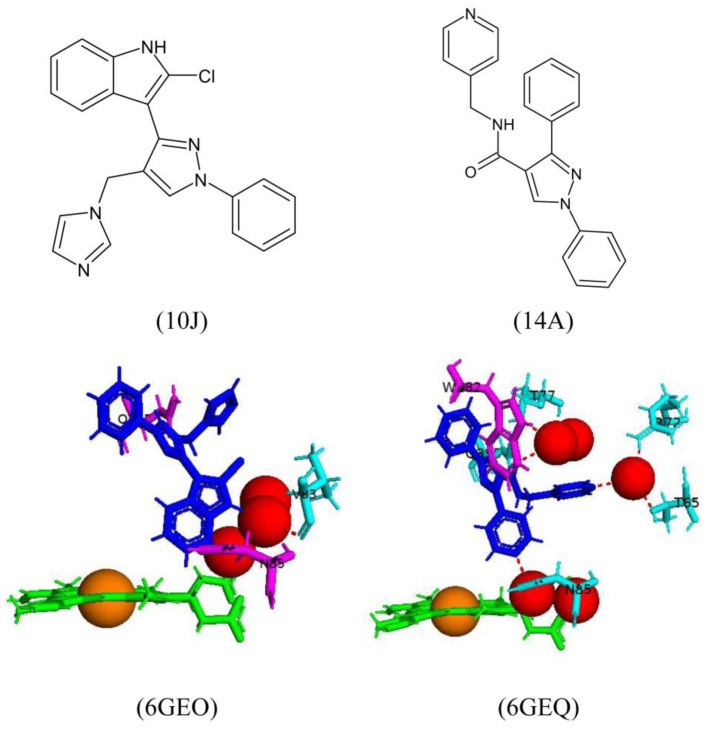
Analysis of CYP121A1 interactions with an indole and 4-pyridyl derivative, respectively. The chemical structures of the compounds are shown in the upper panel, and their interactions with CYP121A1 are shown in the lower panel. Heme is shown in green, iron is shown as a brown sphere, and ligands are shown in blue. Amino acid residues sharing a direct and water-mediated polar interaction with the ligands are shown in cyan, and amino acid residues sharing an arene-hydrogen bond interaction are shown in magenta. Polar interactions are indicated as red dashed lines, water molecules are represented as red spheres, and amino acid residues are labeled according to their single-letter codes. PDB code and derivative number are shown within brackets underneath the respective model and chemical structure. A list of all amino acid residues within 5 Å of the ligands is shown in Table 2.

Two biaryl derivatives were successfully crystallized with CYP121A1 [32], namely an indole derivative (10J) and an extended 4-pyridyl derivative (14A) (Figure 18). 10J formed a direct hydrogen bond with one water molecule and a water-mediated bond with Val83 (Figure 18). Two arene–H interactions were formed between the imidazole and Gln385 and the indole benzene ring and Asn85 (Figure 18). 14A formed direct hydrogen bonds between the carbonyl oxygen and Gln385, and water-mediated bonds with Thr77 (Figure 18). Both 4-pyridyl groups formed water-mediated hydrogen bonds with Thr65, Arg72, and Asn85 (Figure 18). The benzene ring forms an arene-H interaction with Trp182 (Figure 18).

Comparative molecular analysis between fluconazole, cYY, and 10J revealed all compounds interact either directly or indirectly with amino acids Met62, Val83, and Asn85. In particular, hydrogen-bonding interactions with Gln385 (10J and fluconazole) or Arg386 (cYY) would appear to effectively block the access channel to the CYP121A1 active site and thus inhibit this enzyme functionality [32].

### 2.10. Direct Coordination with the Sixth Water Ligand Could Be a Strategy to Inhibit CYP121A1

cYY substrate orientation within the active site of CYP121A1 revealed a hydrogen-bonded network involving the sixth water ligand. As previously mentioned, many compounds are known to coordinate with the heme iron indirectly via a water molecule. Various compounds were screened to investigate the consequences of directly interacting with the sixth water ligand in CYP121A1. One compound in particular, I:47, was considered a potent inhibitor of CYP121A1 [33]. I:47 consists of an imidazole moiety, a propylene linker, and a methylene bridge connecting the ring to the two aromatic moieties [33]. I:47 showed a high binding affinity to CYP121A1, biological activity towards *M. tuberculosis*, and it had a molecular structure similar to that of the native substrate [33].

**Figure 19:**
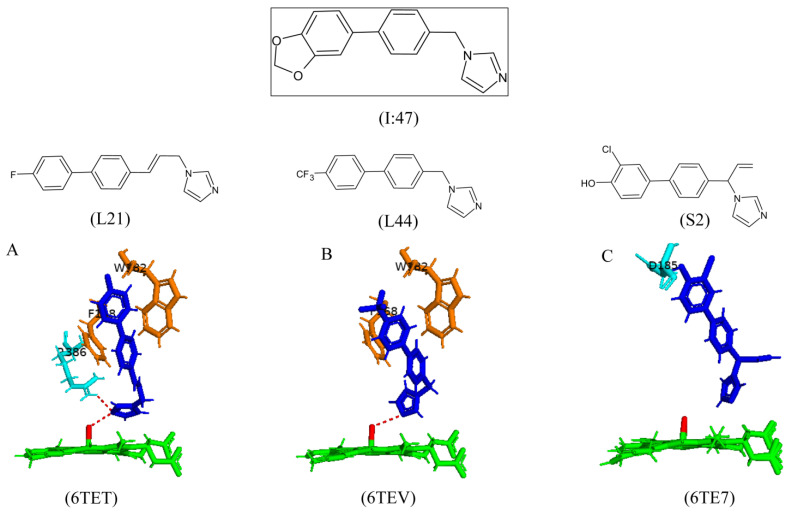
Analysis of CYP121A1 interactions with potential inhibitors. The parent compound is shown within a box. The chemical structures of the compounds are shown in the upper panel, and their interactions with CYP121A1 are shown in the lower panel. (**A**) represents L21, (**B**) represents L44, and (**C**) represents S2. Heme is shown in green, iron is shown as a red stick, and ligands are shown in blue. Amino acid residues sharing a direct and water-mediated polar interaction with the ligands are shown in cyan, and amino acid residues sharing a hydrophobic interaction are shown in orange. Polar interactions are indicated as red dashed lines, and amino acid residues are labeled according to their single-letter codes. PDB codes and analog numbers are shown within brackets underneath the respective model and chemical structure. A list of all amino acid residues within 5 Å of the ligands is shown in Table 2.

Various substrate analogs were created and bound to CYP121A1 in order to further investigate the role(s) of this compound’s different chemical moieties and the consequence of their substitution. Three compounds were successfully crystallized in complex with CYP121A1, namely, L21 (6TET), L44 (6TEV), and S2 (6TE7) [34]. The aromatic side chain of L21 was substituted with fluorine and has an ethenyl-substituted methylene bridge (Figure 19) [34]. The aromatic side chain was substituted with trifluoromethyl in the L44 compound, and the methylene bridge from I:47 was retained (Figure 19) [34]. However, in the S2 analog, the methylene bridge was substituted with ethylene, and the aromatic side chain was substituted at two positions with one hydroxyl group and one chlorine atom (Figure 19) [34].

L21 was bound within a substrate access channel in CYP121A1, with the imidazole moiety directly facing the heme iron (Figure 19A). The nitrogen atom of the imidazole moiety forms a direct hydrogen bond with the sixth water ligand and Arg386 (Figure 19A). The aromatic side chains form hydrophobic interactions with Phe168 and Trp182 (Figure 19A) [34]. L44 bound similarly to L21, with the imidazole moiety again facing towards the heme and sharing a direct hydrogen bond with the sixth water ligand (Figure 19B). Due to a shorter bridge, the two phenyl moieties shifted their position compared to L21, and this resulted in less-than-optimal hydrophobic interactions with Phe168 and Trp182 (Figure 19B) [34]. Interestingly, the combination of a shorter bridge and the orientation of the two phenyl rings in the S2 analog resulted in the compound being trapped in the active site of CYP121A1, as there was a direct hydrogen bond between the hydroxyl group and Asp185 (Figure 19C) [34]. This prevented the compound from forming a hydrogen bond with the sixth water ligand and it being able to be displaced by cYY, as this compound will not prevent the hydrogen-bonding network.

Two of the three compounds shared a direct hydrogen bond with the sixth water ligand and could disrupt the hydrogen bonding network required for cYY binding. The presence of these inhibitors should inhibit the enzymatic conversion of the native substrate. Indeed, in vitro reconstitution assays revealed that L21 inhibited the CYP121A1 substrate conversion at certain concentration levels [34]. However, further studies will need to be carried out in order to analyze its inhibitory effects on the growth of *M. tuberculosis*.

### 2.11. Compounds in the Distal Region of the Heme May Disrupt the Hydrogen-Bonded Network of CYP121A1

A series of D-tryptophan triazole derivatives showed relative potency against CYP121A1 in a spectrophotometric assay [35]. However, none of these compounds were crystallized in complex with CYP121A1; so, their binding mode(s) could not be scrutinized. Further chemical modifications were performed on this series of compounds as a route to developing a specific CYP121A1 inhibitor. The triazole ring was replaced with a pyrimidine ring to improve water solubility [36]. The lipophilicity of the compounds was reduced by replacing unsaturated aromatic sidechains with achiral saturated rings containing solubilizing or heavier heteroatoms such as sulfur [36].

**Figure 20:**
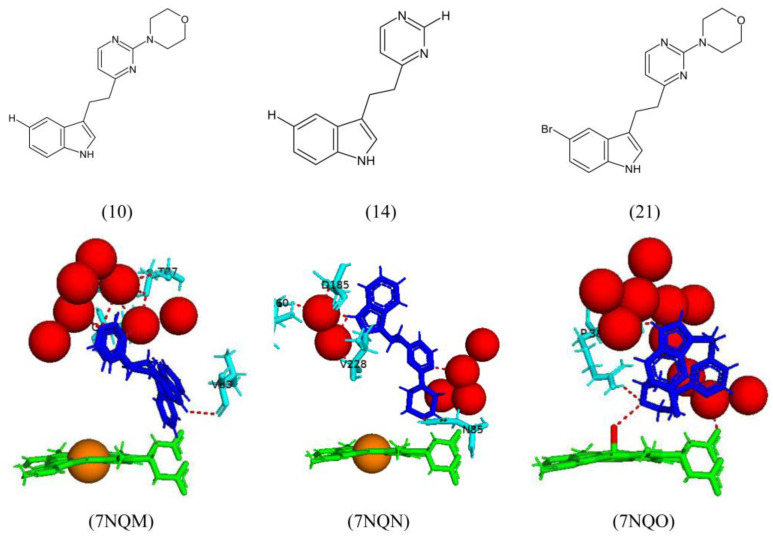
Analysis of CYP121A1 interactions with potential inhibitors. The chemical structures of the compounds are shown in the upper panel, and their interactions with CYP121A1 are shown in the lower panel. Heme is shown in green, iron is shown as a red stick or brown sphere, and ligands are shown in blue. Amino acid residues sharing a direct and water-mediated polar interaction with the ligands are shown in cyan. Polar interactions are indicated as red dashed lines, and amino acid residues are labeled according to their single-letter codes. PDB codes and compound numbers are shown within brackets underneath the respective model and chemical structure. A list of all amino acid residues within 5 Å of the ligands is shown in Table 2.

X-ray crystal structural analysis of compounds 10, 14, and 21 in complex with CYP121A1 were undertaken. However, incomplete ligand electron density was obtained for compounds 10 and 21 [36]. Compound 10 shares a direct hydrogen bond with Val83, three water molecules, and water-mediated hydrogen bonds with Trp77, Ala167, and Gln385 (Figure 20). Compound 21 interacts directly with the sixth water ligand and shares a direct hydrogen bond with Arg386, while water-mediated hydrogen bonds were shared with Gln385 and the heme carboxylate group (Figure 20).

Compound 14 is bound within the CYP121A1 active site in a region distal to the heme cofactor, resulting in no displacement of the sixth water ligand. Interestingly, compound 14 does not interact at all with the heme iron via a water molecule (Figure 20). A direct hydrogen bond is formed between the compound and Asn85; this bond is also observed between cYY and CYP121A1. cYY is surrounded by water molecules within the CYP121A1 active site; upon binding of compound 14, the indole moiety disrupts the hydrogen-bonded network and results in a large number of water molecules being expelled from the active site (Figure 20) [36]. Compound 14 showed a novel binding mode distal to the heme cofactor and could still disrupt the hydrogen bonding network. This may present a new starting point for further anti-TB P450 drug development.

## 3. Materials and Methods

### 3.1. Retrieving of CYP121A1 Member’s Structures

Fifty-three CYP121A1 protein crystal structures available for public use at the Research Collaboratory for Structural Bioinformatics Protein Data Bank (RCSB PDB) [20] were used in this study (Table S2).

### 3.2. CYP121A1 Active Site Analysis

CYP121A1 active site analysis was performed following methodology recently developed and described in our laboratory [19]. Individual CYP121A1 crystal structures were analyzed and assigned to either an open conformation (containing only heme cofactor) or a closed conformation (bound with a chemical as containing heme). Each CYP121A1 crystal structure’s area and volume were analyzed for both open and closed conformations using the Computed Atlas of Surface Topography of Proteins (CASTp) database [37]. For active site analysis, each PDB file was individually uploaded onto PyMOL software, Version 2.2.5 [38]. The active site cavities were selected using heme as the center point of the binding pocket, and amino acid residues within 5 Å were chosen. The amino acid residues were recorded for both open and closed conformations. Consequently, conserved and critical amino acids within the open and closed conformation were identified. The active site was represented by the heme in open conformation and the heme and other ligands in closed conformation within the binding pocket. The amino acid residues were represented as sticks and labeled using the one-letter amino acid codes.

### 3.3. Analysis of Ligand Interactions in Closed Conformation PDB Files Using PyMOL

In total, 44 of the 53 crystal structures studied were in the closed conformation. Firstly, individual PDB files were uploaded onto PyMOL, and the active site cavity was selected, as mentioned in Section 3.2. If the bound ligand extended out of the selected binding pocket, 5 Å from the ligand was chosen to include all interacting amino acid residues. The amino acids were represented as sticks and labeled according to their one-letter amino acid code. Polar contacts with any atoms were then selected. If ligand interactions with specific amino acid residues were determined, dashed lines would appear connecting the ligand and the specific amino acid residue, water molecule, or solvent molecule. If the ligand interacted with a water molecule, polar interactions with the water molecule were selected to analyze water-mediated bonds with the ligand. For data obtained from the published literature, hydrophobic residues within 5 Å were selected and shown as sticks. The amino acid residues that were not interacting with the ligand were removed.

### 3.4. Annotation of P450 Characteristic Secondary Structures and Substrate Recognition Sites (SRSs)

**Figure 21:**
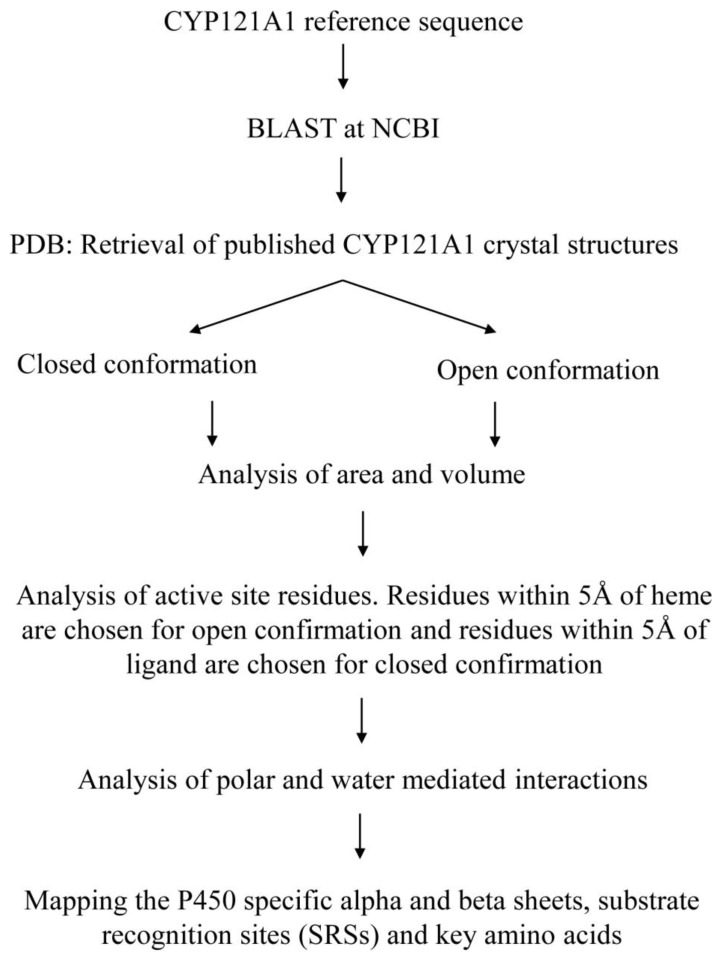
The flow-chart graphic depicts the general methodology used in this investigation.

Descriptions of P450 characteristics and identities for CYP121A1 alpha helices and beta sheets were carried out as described elsewhere [39]. The CYP121A1 PDB file (1N4G) was chosen and uploaded onto PyMOL software, Version 2.2.5 [39]. The alpha helices and beta sheets were assigned different colors, which were used to map the secondary structural elements of the chosen protein sequence. Subsequently, alpha helices and beta sheets were named following the work in [39]. The identification of CYP121A1 substrate recognition sites (SRSs) was carried out as described elsewhere [40]. Briefly, SRS1 was mapped between alpha helix B and C along the BC-loop, SRS2 was located in the C-terminal end of alpha helix F, and SRS3 and SRS4 were located by the N-terminal regions of alpha helix G, SRS5 was found within beta sheet 1–4, and SRS6 was located in beta-sheet 4–1. Figure 21 shows a flow-chart diagram that summarizes the strategies utilized in this investigation.

## 4. Conclusions

**Figure 22:**
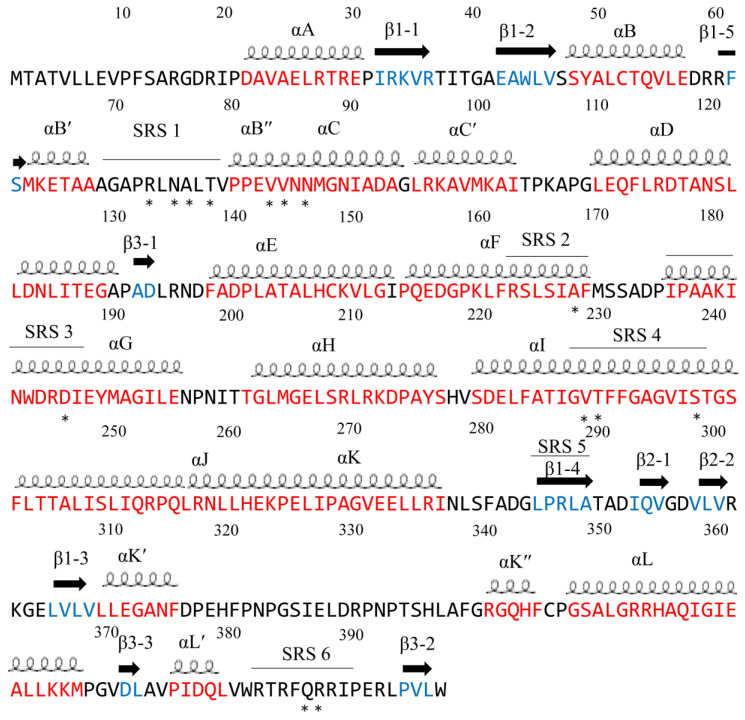
P450 characteristics’ secondary structural analysis of CYP121A1. Alpha helices are shown as coils, the amino acid residues part of alpha helices is highlighted in red, beta sheets are shown as black arrows, and the amino acid residues part of beta sheets is highlighted in blue. Amino acid residues involved in direct polar interactions with ligands are represented as (*) underneath the respective residue. P450 characteristic naming for alpha helices, beta sheets, and identifying substrate recognition sites (SRS) was performed following the methods described elsewhere [39,40].

CYP121A1 is highly substrate-specific, and the molecular basis of this catalytic specificity can be attributed to the control of ligand binding, which results from both restricted binding specificity and a fine-tuned P450-substrate relationship. Further analysis revealed that the CYP121A1 active site is highly rigid, and the binding of molecules within the active site does not significantly change the area and volume. Understanding how substrates and inhibitors gain access into CYP121A1 active site may be further illuminated using molecular dynamic simulation approaches. Comparing ligand binding to ligand-free CYP121A1 crystal structures may reveal exact geometries and conformational changes that occur on ligand binding, including information on ligand recognition and regio- and stereo-specificity at the atomic level. Such work can help in rational drug design by providing experimentally testable models that may contribute to the generation of novel anti-TB drugs. A closer examination of the CYP121A1 active site revealed various amino acid residues involved in a hydrogen bonding network above the heme that play a role in the catalytic reaction mechanism. cYY is a Y-shaped compound, and thus, Y-shaped inhibitors were designed and blocked the substrate access channel of CYP121A1, preventing cYY from entering the active site. Fragment-based design of inhibitor studies revealed that interactions with the heme could be a potential starting point for inhibitor designs, as direct interaction with the heme iron led to disrupting the catalytic mechanism of CYP121A1. Introducing halogens that interact with the heme group is also a feasible approach to developing next-generation, tight-binding inhibitors of CYP121. However, potential inhibitors that interact with the sixth water ligand are also able to disrupt the hydrogen bonding network, and inhibitors containing an indole moiety bound in the distal region of the heme led to a large number of water molecules being expelled out of the active site and thus disrupting the hydrogen bonding network. It is noteworthy that amino acids involved in polar interactions with ligands can be found in only four out of the six substrate recognition sites (SRSs) and between the αB″ and αC helices (Figure 22).

This study provides comprehensive information on mycobacterial CYP121A1 structure–function analysis and highlights the active site cavity dynamics and amino acids’ role in catalysis. This work will further contribute to the design of new and specific CYP121A1 inhibitors that will ultimately be developed into novel anti-TB drugs. Such work will be developed in our laboratories going forward.

## Figures and Tables

**Table 1:** Analysis of amino acid dynamics for CYP121A1. Amino acid residues within 5 Å of the heme were selected. Amino acids that are conserved, unique, and part of the active site were presented.

Conformation	Number of Crystal Structures	Amino Acids Part of the Active Site (Number of Amino Acids)	Conserved Amino Acid Residues (Number of Amino Acids)	Unique Amino Acids (Number of Amino Acids)
Open	9	Leu55, Met62, Val83, Asn85, Met86, Ile102, His146, Thr229, Phe230, Ala233, Gly234, Ser237, Thr238, Phe241, Leu274, Asn277, Ser279, Phe280, Leu284, Pro285, Arg286, Leu309, Leu336, Ala337, Phe338, Gly339, Gln342, His343, Phe344, Cys345, Pro346, Gly347, Ser348, Leu350, Gly351, Arg386 (36)	Leu55, Met62, Asn85, Met86, Ile102, His146, Phe230, Ala233, Gly234, Ser237, Thr238, Phe241, Leu274, Asn277, Ser279, Phe280, Leu284, Pro285, Arg286, Leu309, Leu336, Ala337, Phe338, Gly339, Gln342, His343, Phe344, Cys345, Pro346, Gly347, Leu350, Gly351, Arg386 (33)	None
Closed	44	Leu55, Met62, Val82, Val83, Asn85, Met86, Ile89, Arg95, Ile102, Leu145, His146, Val149, Leu150, Thr229, Phe230, Ala233, Gly234, Val235, Ser237, Thr238, Phe241, Leu274, Asn277, Ser279, Phe280, Leu284, Pro285, Arg286, Leu309, Leu336, Ala337, Phe338, Gly339, Arg340, Gln342, His343, Phe344, Cys345, Pro346, Gly347, Ser348, Leu350, Gly351, Arg352, His354, Ala355, Arg386 (47)	Met62, Met86, Ile102, His146, Phe230, Ala233, Gly234, Ser237, Thr238, Phe241, Leu274, Phe280, Leu284, Arg286, Leu309, Leu336, Ala337, Phe338, Gly339, Gln342, His343, Phe344, Cys345, Pro346, Gly347, Leu350, Gly351 (27)	Val82, Ile89, Arg95, Leu145, Val149, Leu150, Val235, Arg340, Arg352, His354, Ala355 (11)

**Table 2:** List of amino acid residues within 5 Å of the ligand of CYP121A1 proteins. Amino acid residues, shown in bold, share a direct polar interaction with the ligand.

PDB Code	Amino Acid Residues
1N4G	Thr77, Val78, Ala167, Phe168, Trp182, Val228, Thr229, Gly232, Ala233, Gly234
2IJ7 (B)	Met62, **Thr77**, Val78, Val82, Val83, Asn84, Asn85, Met86, Ala167, Phe168, **Thr229**, Ala233, Ser237, Phe280, Gln385, Arg386
2IJ7 (C)	Met62, **Thr77**, Val78, Val82, Val83, Asn84, Asn85, Met86, Ala167, Phe168, **Thr229**, Ala233, Ser237, Phe280, Gln385, Arg386
2IJ7 (D)	Met62, **Thr77**, Val78, Val82, Val83, Asn84, Asn85, Met86, Ala167, Phe168, Thr229, Ala233, Ser237, Phe280, **Gln385**, **Arg386**
2IJ7 (A)	Met62, **Thr77**, Val78, Val82, Val83, Asn84, Asn85, Met86, Ala167, Phe168, Thr229, Ala233, Ser237, Phe280, **Gln385**, Arg386
2IJ7 (E)	Met62, **Thr77**, Val78, Val82, Val83, Asn84, Asn85, Met86, Ala167, Phe168, Thr229, Ala233, Ser237, Phe280, Gln385, Arg386
3G5H	Met62, Thr77, Val78, Val82, Val83, **Asn85**, Ala167, Phe168, Trp182, Val228, Thr229, Gly232, Ala233, Phe280, Gln385, **Arg386**
4G1X	Met62, **Asn85**, Thr229, Phe230, Ala233, Ser237, Phe280, Cys345, Arg386
4G2G	**Thr77**, Val78, Val82, **Asn85**, Leu164, Ala167, Phe168, Trp182, Asp185, Val228, Thr229, Gly232, Ala233, **Gln385**
4G44	**Asn85**, Thr229, Phe230, Ala233, **Ser237**, Phe280, Cys345, Arg386
4G45	Met62, Val83, Ala233, **Ser237**, Phe280, Cys345, Arg386
4G46	**Thr77**, Val78, Val82, Val83, Ala167, Phe168, Trp182, Val228, Thr229, **Gln385**
4G47	**Thr77**, Val78, Val82, **Asn85**, Ser163, Leu164, Ala167, Phe168, Trp182, Asp185, Val228, Thr229, Gly232, Ala233, **Gln385**
4G48	**Thr77**, Val78, Val82, Val83, Asn85, Leu164, **Ala167**, Phe168, Trp182, **Asp185**, **Val228**, Thr229, Gly232, Ala233, Gln385
4ICT	Met62, Thr77, Val78, Val82, Val83, **Asn85**, Ala167, Phe168, Trp182, Val228, Thr229, Gly232, Ala233, Gln385, Arg386
4IPS	Met62, Thr65, Leu73, Asn74, Thr77, Val78, Val82, Val83, **Asn85**, Met86, Ala167, Phe168, Trp182, Val228, Thr229, Gly232, Ala233, Pro285, Gln385
4IPW	Met62, **Thr77**, Val78, Val82, Val83, **Asn85**, **Ala167**, Phe168, Trp182, Val228, Thr229, Gly232, Ala233, Phe280, Gln385, Arg386
4IQ7	Met62, Thr77, Val78, Val82, Val83, **Asn85**, Met86, Ala167, Phe168, Trp182, Val228, Thr229, Gly232, Ala233, Ser237, Phe280, Gln385, **Arg386**
4IQ9	Met62, Thr77, Val78, Val82, **Val83**, **Asn85**, Ala167, Phe168, Trp182, Val228, Thr229, Gly232, Ala233, Ser237, Phe280, Gln385, **Arg386**
5EDT	**Thr77**, Val78, Leu159, Ser163, Ala167, Phe168, **Ala178**, Asn181, Trp182, **Asp185**, Val228, Thr229, Gly232, Ala233
5IBD	Met62, Leu76, Thr77, Val78, Val82, Val83, Asn85, Ala167, Phe168, Trp182, Val228, Thr229, Gly232, Ala233, **Ser237**, Phe280, Cys345, Gln385, Arg386
5IBE	Met62, Leu76, Thr77, Val78, Val82, Val83, Asn85, Ala167, Phe168, Trp182, Thr229, Ala233, **Ser237**, Phe280, Cys345, Gln385, Arg386
5IBF1	Met62, Leu76, Val78, Val82, Val83, Asn85, Thr229, Ala233, **Ser237**, Phe280, Cys345, Arg386
5IBF2	Thr77, Val78, Ser163, Leu164, Ala167, Phe168, Ala178, Lys179, Asn181, Trp182, Asp185, Val228, Thr229, Gly232, Ala233, Gln385
5IBG1	**Thr77**, Val78, Val82, Leu164, Ala167, Phe168, Trp182, **Val228**, Thr229, Gly232, Ala233, **Ser237**, Phe280, Cys345, **Gln385**, Arg386
5IBG2	Leu76, Val78, Pro79, Pro80, Ala178, Lys179, Trp182, Asp183
5IBH	**Asn74**, **Ala75**, Leu76, **Thr77**, Val78, Val82, Val83, **Asn85**, Leu164, Ala167, Phe168, Trp182, **Asp185**, Val228, Thr229, Gly232, Ala233, Gln385
5IBI	Thr65, **Arg72**, Leu76, **Thr77**, Val78, Val82, Val83, **Asn85**, Leu164, Ala167, Phe168, Trp182, Asp185, Val228, Thr229, Gly232, Ala233, Pro285, **Gln385**
5IBJ	Thr77, Val78, Val82, Val83, Asn85, Leu160, Ser163, Leu164, Ala167, Phe168, Asn181, Trp182, Asp185, Val228, **Thr229**, Gly232, Ala233
5OP9	Met62, Ala75, Leu76, Thr77, Val78, Val82, Val83, Asn85, Met86, Ser163, Leu164, Ala167, Phe168, Trp182, Asp185, Val228, Thr229, Gly232, Ala233, Gln385
5OPA	Thr77, Val78, Val82, Val83, Asn85, Met86, Ser163, Leu164, Ala167, Phe168, Trp182, Asp185, Val228, Thr229, Gly232, Ala233, Gln385
6GEO	Met62, Asn74, Ala75, Leu76, Thr77, Val78, Val82, Val83, Asn85, Met86, Ser163, Leu164, Ala167, Phe168, Trp182, Asp185, Val228, Thr229, Phe230, Phe231, Gly232, Ala233, Gln385
6GEQ	Met62, Thr65, Arg72, Leu73, Asn74, Leu76, Thr77, Val78, Val82, Val83, Asn85, Ser163, Leu164, Ala167, Phe168, Ala178, Asn181, Trp182, Asp185, Val228, Thr229, Phe230, Gly232, Ala233, Pro285, **Gln385**
6RQ0	Met62, Thr77, Val78, Val82, Val83, **Asn85**, Ala167, Phe168, Trp182, Val228, Thr229, Gly232, Ala233, Phe280, Gln385, **Arg386**
6RQ1	Met62, Thr77, Val78, Val82, Val83, **Asn85**, Ala167, Phe168, Trp182, Val228, Thr229, Gly232, Ala233, Ser237, Phe280, Gln385, **Arg386**
6RQ3	Met62, Thr77, Val78, Val82, Val83, **Asn85**, Ala167, Phe168, Trp182, Thr229, Ala233, Ser237, Phe280, Gln385, **Arg386**
6RQ5	Met62, Thr77, Val78, Val82, Val83, **Asn85**, Ala167, Phe168, Trp182, Val228, Thr229, Gly232, Ala233, Phe280, Gln385, **Arg386**
6RQ6	Met62, Thr77, Val78, Val82, Val83, **Asn85**, Ala167, Phe168, Trp182, Val228, Thr229, Gly232, Ala233, Phe280, Gln385, **Arg386**
6RQ8	Met62, Thr77, Val78, Val82, Val83, **Asn85**, Ala167, Phe168, Trp182, Val228, Thr229, Gly232, Ala233, Ser237, Phe280, Gln385, **Arg386**
6RQ9	Met62, Thr77, Val78, Val82, Val83, **Asn85**, Ala167, Phe168, Trp182, Val228, Thr229, Gly232, Ala233, Ser237, Phe280, Gln385, **Arg386**
6RQB	Met62, Thr77, Val78, Val82, Val83, **Asn85**, Ala167, Phe168, Trp182, Val228, **Thr229**, Gly232, Ala233, Ser237, Phe280, Gln385, **Arg386**
6RQD	Met62, Thr77, Val78, Val82, Val83, **Asn85**, Ala167, Phe168, Trp182, Val228, **Thr229**, Gly232, Ala233, Ser237, Phe280, Gln385, **Arg386**
6TE7	Thr77, Val78, Val82, Val83, Asn85, Ser163, Leu164, Ile166, Ala167, Phe168, Ala178, Asn181, Trp182, **Asp185**, Val228, Thr229, Gly232, Ala233, Gln385, Arg386
6TET	Met62, Thr77, Val78, Asn85, Ser163, Leu164, Ala167, Phe168, Ala178, Asn181, Trp182, Asp185, Val228, Thr229, Phe230, Gly232, Ala233, Ile236, Ser237, Phe280, Leu284, Gln385, **Arg386**
6TEV	Met62, Thr77, Val78, Asn85, Leu160, Ser163, Leu164, Ala167, Phe168, Ala178, Asn181, Trp182, Asp185, Val228, Thr229, Phe230, Phe231, Gly232, Ala233, Ile236, Ser237, Phe280, Gln385, Arg386
6UPG	Met62, Thr77, Val78, Val82, Val83, **Asn85**, Ala167, Phe168, Trp182, Val228, Thr229, Gly232, Ala233, Ser237, Phe280, Gln385, **Arg386**
6UPI	Met62, Thr77, Val78, Val82, Val83, **Asn85**, Met86, Ala167, Phe168, Trp182, Val228, Thr229, Gly232, Ala233, **Ser237**, Phe280, Gln385, **Arg386**
7NQM	Met62, Asn74, Thr77, Val78, Val82, **Val83**, Asn84, Asn85, Met86, Leu164, Ala167, Phe168, Trp182, Val228, Thr229, Phe230, Gly232, Ala233, Pro285, Arg286, His343, Arg386
7NQN	Met62, Thr77, Val78, Val82, Val83, **Asn85**, Met86, Leu160, Ser163, Leu164, Ile166, Ala167, Phe168, Ala177, Ala178, Lys179, Asn181, Trp182, Asp185, Val228, Thr229, Phe230, Phe231, Gly232, Ala233, Arg386
7NQO	Met62, Thr65, Leu76, Thr77, Val78, Val82, Val83, Asn85, Met86, Ala167, Phe168, Trp182, Val228, Thr229, Phe230, Gly232, Ala233, Ile236, Ser237, Phe280, Leu284, Pro285, Arg286, Gln385, **Arg386**

## Data Availability

Data are contained within this article.

## Supplementary Materials

The following supporting information can be downloaded at: https://www.mdpi.com/article/10.3390/ijms25094886/s1.
