# Supplementary material for: Structure–Function Analysis of the Essential Mycobacterium tuberculosis P450 Drug Target, CYP121A1
Source: Int J Mol Sci. 2024 Apr 30;25(9):4886. doi: 10.3390/ijms25094886 (PMC11084333; doi:10.3390/ijms25094886)
Supplement: Supplementary file 1 [file ijms-25-04886-s001.zip › ijms-2979126-supplementary.pdf]

Article

Structure–Function Analysis of the Essential *Mycobacterium tuberculosis* P450 Drug Target, CYP121A1

Tiara Padayachee [1], David C. Lamb [2], David R. Nelson [3] and Khajamohiddin Syed [1,*]

[1] Department of Biochemistry and Microbiology, Faculty of Science, Agriculture and Engineering, University of Zululand, Empangeni 3886, South Africa; teez07padayachee@gmail.com
[2] Faculty of Medicine, Health and Life Sciences, Swansea University, Swansea SA2 8PP, UK; d.c.lamb@swansea.ac.uk
[3] Department of Microbiology, Immunology and Biochemistry, University of Tennessee Health Science Center, Memphis, TN 38163, USA; drnelson1@gmail.com
* Correspondence: syedk@unizulu.ac.za or khajamohiddinsyed@gmail.com; Tel.: +27-035-902-6857

Table S1: 53 CYP121A1 crystal structures active site cavity area, volume, and conformations.

PDB code	Area (SA) Å^2	Volume (SA) Å^3	Conformation
1N4G	1228	838	Closed
2IJ7	1127	729	Closed
3G5H	1185	738	Closed
4G1X	1150	790	Closed
4G2G	1124	714	Closed
4G44	1136	769	Closed
4G45	1131	744	Closed
4G46	1142	732	Closed
4G47	1140	726	Closed
4G48	1199	761	Closed
4ICT	1166	777	Closed
4IPS	1176	746	Closed
4IPW	1198	758	Closed
4IQ7	1172	744	Closed
4IQ9	1187	771	Closed
5EDT	1146	726	Closed
5IBD	1141	767	Closed
5IBE	1139	738	Closed
5IBF	1005	730	Closed
5IBG	1168	782	Closed
5IBH	1155	728	Closed
5IBI	1226	794	Closed
5IBJ	1186	781	Closed
5OP9	994	703	Closed
5OPA	1133	745	Closed
5WP2	1103	717	Closed
6GEO	1226	784	Closed
6GEQ	1159	751	Closed
6RQ0	1209	778	Closed
6RQ1	1148	738	Closed
6RQ3	1199	766	Closed
6RQ5	1085	690	Closed
6RQ6	1165	751	Closed
6RQ8	1164	742	Closed
6RQ9	1127	739	Closed
6RQB	1150	728	Closed
6RQD	1159	731	Closed
6TE7	1011	698	Closed
6TET	1038	703	Closed
6TEV	1044	712	Closed
6UPG	1103	730	Closed
6UPI	993	699	Closed

7NQM	1093	697	Closed
7NQN	1103	725	Closed
7NQO	1108	725	Closed
1N40	1170	738	Open
2IJ5	1169	777	Open
3CXV	1175	743	Open
3G5F	1158	770	Open
3CY0	1166	732	Open
3CY1	1171	727	Open
3CXX	1163	722	Open
3CXY	1183	782	Open
3CXZ	1215	769	Open

Table S2: CYP121A1 member structures were used in the study. The PDB codes and their references are presented.

PDB code	Reference
1N4G, 1N40	[1]
2IJ7, 2IJ5	[2]
3G5H, 3G5F	[3]
4G1X, 4G2G, 4G44, 4G45, 4G46, 4G47, 4G48	[4]
4ICT, 4IPS, 4IPW, 4IQ7, 4IQ9	[5]
5EDT, 5IBD, 5IBE, 5IBF, 5IBG, 5IBH, 5IBI, 5IBJ	[6]
5OP9, 5OPA	[7]
6GEO, 6GEQ	[8]
6RQ0, 6RQ1, 6RQ3, 6RQ5, 6RQ6, 6RQ8, 6RQ9, 6RQB, 6RQD	[9]
6TE7, 6TET, 6TEV	[10]
6UPG, 6UPI	[11]
7NQM, 7NQN, 7NQO	[12]
3CXV	[13]

References:

1. Leys, D.; Mowat, C.G.; McLean, K.J.; Richmond, A.; Chapman, S.K.; Walkinshaw, M.D.; Munro, A.W. Atomic structure of Mycobacterium tuberculosis CYP121 to 1.06 A reveals novel features of cytochrome P450. *The Journal of biological chemistry* **2003**, *278*, 5141-5147, doi:10.1074/jbc.M209928200.
2. Seward, H.E.; Roujeinikova, A.; McLean, K.J.; Munro, A.W.; Leys, D. Crystal structure of the Mycobacterium tuberculosis P450 CYP121-fluconazole complex reveals new azole drug-P450 binding mode. *The Journal of biological chemistry* **2006**, *281*, 39437-39443, doi:10.1074/jbc.M607665200.
3. Belin, P.; Le Du, M.H.; Fielding, A.; Lequin, O.; Jacquet, M.; Charbonnier, J.B.; Lecoq, A.; Thai, R.; Courcon, M.; Masson, C.; et al. Identification and structural basis of the reaction catalyzed by CYP121, an essential cytochrome P450 in Mycobacterium tuberculosis. *Proc Natl Acad Sci U S A* **2009**, *106*, 7426-7431, doi:10.1073/pnas.0812191106.

4. Hudson, S.A.; McLean, K.J.; Surade, S.; Yang, Y.Q.; Leys, D.; Ciulli, A.; Munro, A.W.; Abell, C. Application of fragment screening and merging to the discovery of inhibitors of the Mycobacterium tuberculosis cytochrome P450 CYP121. *Angewandte Chemie (International ed. in English)* **2012**, *51*, 9311-9316, doi:10.1002/anie.201202544.
5. Fonvielle, M.; Le Du, M.H.; Lequin, O.; Lecoq, A.; Jacquet, M.; Thai, R.; Dubois, S.; Grach, G.; Gondry, M.; Belin, P. Substrate and reaction specificity of Mycobacterium tuberculosis cytochrome P450 CYP121: insights from biochemical studies and crystal structures. *The Journal of biological chemistry* **2013**, *288*, 17347-17359, doi:10.1074/jbc.M112.443853.
6. Kavanagh, M.E.; Coyne, A.G.; McLean, K.J.; James, G.G.; Levy, C.W.; Marino, L.B.; de Carvalho, L.P.; Chan, D.S.; Hudson, S.A.; Surade, S.; et al. Fragment-Based Approaches to the Development of Mycobacterium tuberculosis CYP121 Inhibitors. *Journal of medicinal chemistry* **2016**, *59*, 3272-3302, doi:10.1021/acs.jmedchem.6b00007.
7. Taban, I.M.; Elshihawy, H.; Torun, B.; Zucchini, B.; Williamson, C.J.; Altuwairigi, D.; Ngu, A.S.T.; McLean, K.J.; Levy, C.W.; Sood, S.; et al. Novel Aryl Substituted Pyrazoles as Small Molecule Inhibitors of Cytochrome P450 CYP121A1: Synthesis and Antimycobacterial Evaluation. *Journal of medicinal chemistry* **2017**, *60*, 10257-10267, doi:10.1021/acs.jmedchem.7b01562.
8. Kishk, S.M.; McLean, K.J.; Sood, S.; Smith, D.; Evans, J.W.D.; Helal, M.A.; Gomaa, M.S.; Salama, I.; Mostafa, S.M.; de Carvalho, L.P.S.; et al. Design and Synthesis of Imidazole and Triazole Pyrazoles as Mycobacterium Tuberculosis CYP121A1 Inhibitors. *ChemistryOpen* **2019**, *8*, 995-1011, doi:10.1002/open.201900227.
9. Rajput, S.; McLean, K.J.; Poddar, H.; Selvam, I.R.; Nagalingam, G.; Triccas, J.A.; Levy, C.W.; Munro, A.W.; Hutton, C.A. Structure-Activity Relationships of cyclo(l-Tyrosyl-l-tyrosine) Derivatives Binding to Mycobacterium tuberculosis CYP121: Iodinated Analogues Promote Shift to High-Spin Adduct. *Journal of medicinal chemistry* **2019**, *62*, 9792-9805, doi:10.1021/acs.jmedchem.9b01199.
10. Walter, I.; Adam, S.; Gentilini, M.V.; Kany, A.M.; Brengel, C.; Thomann, A.; Sparwasser, T.; Köhnke, J.; Hartmann, R.W. Structure-Activity Relationship and Mode-Of-Action Studies Highlight 1-(4-Biphenylylmethyl)-1H-imidazole-Derived Small Molecules as Potent CYP121 Inhibitors. *ChemMedChem* **2021**, *16*, 2786-2801, doi:10.1002/cmdc.202100283.
11. Nguyen, R.C.; Yang, Y.; Wang, Y.; Davis, I.; Liu, A. Substrate-Assisted Hydroxylation and O-Demethylation in the Peroxidase-like Cytochrome P450 Enzyme CYP121. *ACS catalysis* **2020**, *10*, 1628-1639, doi:10.1021/acscatal.9b04596.
12. Frederickson, M.; Selvam, I.R.; Evangelopoulos, D.; McLean, K.J.; Katariya, M.M.; Tunnicliffe, R.B.; Campbell, B.; Kavanagh, M.E.; Charoensutthivarakul, S.; Blankley, R.T.; et al. A new strategy for hit generation: Novel in cellulo active inhibitors of CYP121A1 from Mycobacterium tuberculosis via a combined X-ray crystallographic and phenotypic screening approach (XP screen). *European journal of medicinal chemistry* **2022**, *230*, 114105, doi:10.1016/j.ejmech.2022.114105.
13. McLean, K.J.; Carroll, P.; Lewis, D.G.; Dunford, A.J.; Seward, H.E.; Neeli, R.; Cheesman, M.R.; Marsollier, L.; Douglas, P.; Smith, W.E.; et al. Characterization of active site structure in CYP121. A cytochrome P450 essential for viability of Mycobacterium tuberculosis H37Rv. *The Journal of biological chemistry* **2008**, *283*, 33406-33416, doi:10.1074/jbc.M802115200.
